# Disruption of sulfur transferase complex increases bacterial intramacrophage persistence

**DOI:** 10.1371/journal.ppat.1013136

**Published:** 2025-05-14

**Authors:** Huang Tang, Zuoqiang Wang, Congcong Li, Jingchen Yu, Wanqiu Huang, Tao Zhou, Chuanzhen Zhang, Bingjie Wen, Chengyue Wang, Xiaocen Zhu, Danni Wang, Jing Tao, Jie Lu, Jinjing Ni, Yu-Feng Yao

**Affiliations:** 1 Laboratory of Bacterial Pathogenesis, Shanghai Institute of Immunology, Shanghai Jiao Tong University School of Medicine, Shanghai, China; 2 Shanghai Public Health Clinical Center, Fudan University, Shanghai, China; 3 Core Facility of Basic Medical Sciences, Shanghai Jiao Tong University School of Medicine, Shanghai, China,; 4 Department of Infectious Diseases, Ruijin Hospital, Shanghai Jiao Tong University School of Medicine, Shanghai, China; 5 Laboratory of Bacterial Pathogenesis, Department of Microbiology and Immunology, Shanghai Jiao Tong University School of Medicine, Shanghai, China; 6 State Key Laboratory of Microbial Metabolism, and School of Life Sciences and Biotechnology, Shanghai Jiao Tong University, Shanghai, China; 7 Shanghai Key Laboratory of Emergency Prevention, Diagnosis and Treatment of Respiratory Infectious Diseases, Shanghai, China; Universite Paris Descartes Faculte de Medecine, FRANCE

## Abstract

Bacterial persisters contribute significantly to clinical treatment failure and relapse. These cells could resist antibiotic treatment *via* transient phenotypic and gene expression alterations. We conducted a high-throughput screening of *Salmonella* Typhimurium transposon mutants to identify key genes for intramacrophage antibiotic persistence. The results show that a sulfur transferase complex encoded by *yheM*, *yheL, yheN*, *trmU* and *yhhP* are involved in bacterial intramacrophage antibiotic persistence. *Salmonella* could persist in macrophages by downregulating the expression of the sulfur transferase complex during exposure to high concentrations of antibiotics, and even in a persistent infection mouse model. Mechanistically, deletion of *yheM* increases reactive nitrogen species (RNS) in the exponential phase, which inhibits bacterial respiration and ATP generation. In contrast, absence of *yheM* promotes persister formation by elevating (p)ppGpp levels in the stationary phase. Taken together, our data demonstrate that bacteria use the sulfur transferase to coordinate intramacrophage replication and persistence for adaptation to various environmental stresses. These findings reveal the role of the sulfur transferase complex in bacterial intramacrophage persistence and provide a promising target for antibacterial infection therapy.

## Author summary

Numerous bacteria possess the ability to establish long-term persistence within host. The bacterial persistence poses a significant challenge in the clinical management of chronic infections, primarily due to the existence of a small subpopulation of cells that can withstand conventional antimicrobial treatments. In this study, we identify key genes associated with persister formation in response to antibiotics in macrophages using a *Salmonella* transposon mutant library. The results show that several key pathways including the sulfur transfer system, carbon metabolism, cofactors biosynthesis and the type III secretion system are involved in bacterial persistence. Furthermore, we show that the sulfur transferase complex is a key factor in bacterial persistence. Disruption of this sulfur transferase complex increases RNS during the exponential phase and promotes persistence formation by activating RelA to increase (p)ppGpp synthesis during the stationary phase. Overall, deficiency of this sulfur transferase complex increases bacterial persistence both *in vitro* and *in vivo*. Our data demonstrate that the sulfur transferase complex could reprogram bacterial metabolism to modulate persistence and suggest the potential of the sulfur transferase complex as an antibacterial target.

## Introduction

Failure of antibiotic treatment is a major challenge in clinical medicine, mainly due to the widespread use of antibiotics, which exerts increasing selection pressure on bacteria. Many pathogens have developed strategies to survive in both natural and clinical environments when exposed to antibiotics. Pathogens can respond to antibiotics through three main phenomena: resistance, tolerance, and persistence [1–3]. To prevent the growth of resistant bacteria, a much higher minimum inhibitory concentration (MIC) of the antibiotic is required than for susceptible bacteria. Persistence and tolerance do not show an increase in MIC, and their offsprings are still predominantly antibiotic sensitive [1,4,5]. Persistence is a specific type of tolerance, involving a small subpopulation of bacteria that survives antibiotic treatment more effectively than the majority, as evidenced by the biphasic killing curve, which shows a two-phase decline in bacterial numbers [5,6]. Persistence allows bacteria to survive antibiotic treatment both *in vitro* and in the host environment, and to resume growth after antibiotic withdraw [5,7,8]. Persisters are an unstable, non-growing, multidrug-resistant subgroup of cells and are thought to be selected in patients with recurrent infections and antibiotic treatment failure [9,10]. Current studies of persistence focus primarily on *in vitro* conditions, and several mechanisms have been proposed. These include ATP levels affecting cellular respiration and electron transfer [11], the guanosine pentaphosphate ((p)ppGpp) stringent response pathway involved in cellular metabolism and biofilm formation [12,13], SOS reactions related to DNA damage and repair [4], toxin-antitoxin system leading to cell poisoning [14–16], and protein aggregation affecting cellular dormancy [17]. However, the optimized growth conditions of the culture medium cannot fully simulate the growth status of pathogens *in vivo*, which limits the insights into the formation of persistent bacteria during infections [18].

Persisters in clinical treatment complicates efforts to eradicate infections. In addition to challenges such as poor antibiotic permeability in certain tissues and biofilms, persistent infections are thought to be sustained in part by antibiotic persistence [19,20]. Critically, the lifecycle of persisters involves three dynamically regulated phases: (1) formation under antibiotic or host-induced stress, (2) maintenance of a dormant yet viable state during prolonged treatment, and (3) regrowth upon cessation of therapeutic pressure [21–23]. Persisters can endure within infected tissues and cells during antibiotic exposure, and remain dormant. When antibiotic treatment is discontinued, persisters subsequently resuscitate when antibiotic concentrations drop below a threshold. This triphasic factors comprising formation, survival, and resurgence enables bacterial populations to evade eradication, ultimately leading to treatment failure [24]. Several bacterial species, including those causing severe infections, have been observed to form persisters within host cells [25–30]. Macrophages are pivotal immune cells responsible for detecting and eliminating invading microorganisms. Several studies have revealed the presence of bacteria that maintain survival in macrophages under antibiotic treatment, demonstrating that these immune cells are facilitators of persister formation [25,31]. For example, persisters are susceptible to macrophage-induced accumulation of dsDNA breaks, but are also protected by their own RecA-mediated DNA repair, which contributes to their survival during antibiotic exposure [32]. Additionally, persisters can secrete effectors to switch macrophages from a pro-inflammatory to an anti-inflammatory state, thereby creating a more suitable niche for sustained survival [31,33].

Recently, several groups have reported that *Salmonella* can generate non-replicating persisters after ingestion by macrophages located in Peyer’s patches, mesenteric lymph nodes, spleen, and liver [25,31,34,35]. These persister cells are tolerant to antibiotic treatment, and some of them can regrow once the antibiotic pressure is released [36–38]. The heterogeneity of the internal environment of macrophages and bacteria themselves leads to non-growing or the formation of non-replicating cells, which may contribute to persister formation [31]. However, the specific genes and pathways that involved in persister formation within macrophages under antibiotic treatment remain largely unknown.

To investigate the mechanisms of persister formation in *Salmonella* during infection, we used a saturated transposon mutant library to infect macrophages in combination with antibiotic treatment. This assay could identify the gene involved in persister formation in response to antibiotics in macrophages. Our results showed that sulfur transferase genes are involved in persister formation at different stages by mediating the bacterial metabolic network. These findings reveal the role of sulfur transferase in the development of persisters and provide a potential target for novel anti-persister therapies.

## Results

### Tn-seq screening of key genes for antibiotic persistence of *Salmonella* in macrophages

**Fig 1:**
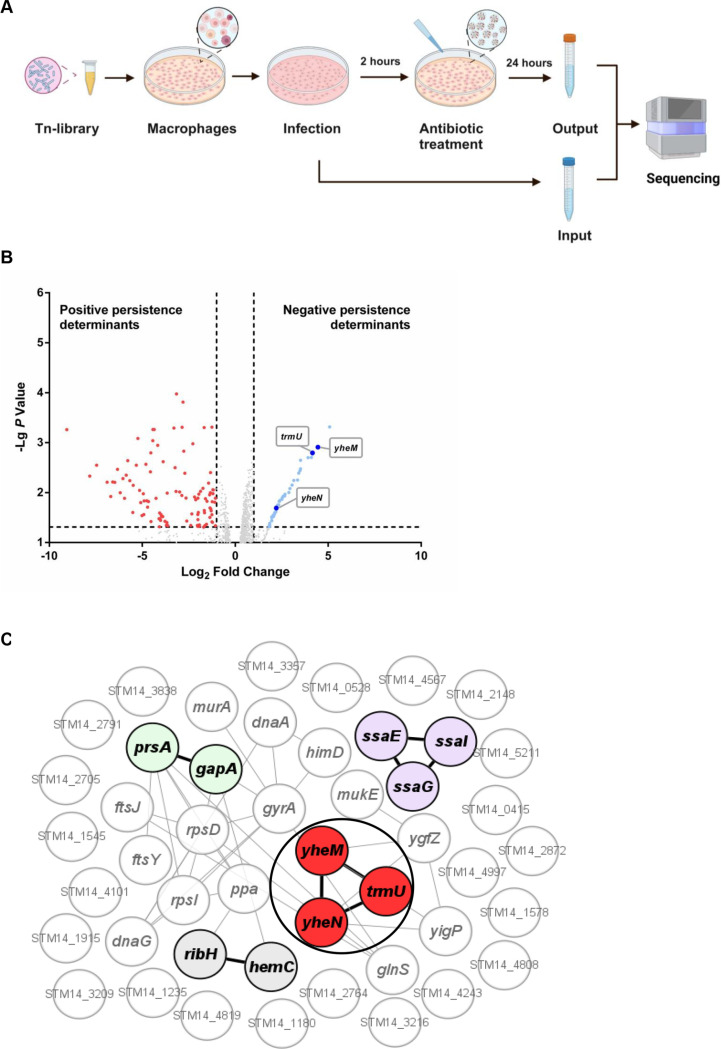
Tn-seq screening of key genes for intramacrophage persistence. (A) Schematic representation of the Tn-seq performed in the model cell lines used in this study. RAW 264.7 cells were infected by 5 × 10^7^ CFUs of the mutant library. Bacterial cells were harvested after antibiotic treatment. Created in BioRender. Tang, H. (2025) https://BioRender.com/r03v702. (B) Volcano plots of Tn-seq screening results display statistical significance against persistence determinants of *S.* Typhimurium transposon mutant in macrophages combined with cefotaxime treatment. Positive persistence determinants are colored red, and negative persistence determinants are colored blue, and sulfur transferase genes, including *yheM, yheN* and *trmU* are highlighted. (C) Associations between negative persistence determinants based on STRING. Genes are shown as circles. The sulfur transferases are colored red and circled, the carbon metabolism genes are colored green, the biosynthesis of cofactors genes are colored gray and the type III secretion system genes are colored purple. Gene interactions are indicated by the gray line, and the interactions between colored genes are highlighted with black lines.

To identify genes that contribute to persister formation in macrophages, we used a saturated transposon mutant library of *Salmonella* [39] to infect mouse macrophage RAW 264.7 cells. After 2 hours of infection, extracellular bacteria were eliminated by gentamicin treatment. One third of the intracellular bacteria were harvested for DNA extraction as the input pool, while the remaining intracellular bacteria were treated with cefotaxime, a β-lactam antibiotic effective against intracellular bacteria [7]. After 24 hours, survived intramacrophage bacteria were isolated, and their genomic DNA was extracted for high-throughput sequencing (Fig 1A). The distribution and abundance of transposon insertion sites in the input and output pools were determined. The relative abundance of each gene in the output pool to the input pool (fold change, FC) was calculated. The final data are summarized in S1 Dataset.

We identified a total of 157 genes involved in persister formation in macrophages. Eighty-eight genes are negative determinants in persister formation, and 105 genes are positive determinants in persister formation (Fig 1B). Next, STRING analysis showed that several members of the metabolism network (sulfur transfer pathway and carbon metabolism), cofactors biosynthesis and the type III secretion system were involved in negative persistence determinants (Fig 1C). In our previous study, we used the transposon mutant library to investigate the stress adaptation mechanism and identified *yheM* as fitness determinant in all 17 stress conditions. Specially, deletion of *yheM* impairs *Salmonella* intramacrophage replication [39]. However, deletion of *yheM* could increase *Salmonella* survival in macrophages in combination with cefotaxime treatment (Fig 1B). Interestingly, Tn-seq results showed that deletion of any sulfur transferase complex genes, including *yheM*, *yheN*, and *trmU* increased bacterial survival in macrophages in combination with cefotaxime treatment. The above genes encode a sulfur transferase complex responsible for the 2-thiolation of 5-methylaminomethyl-2-thiouridine (mnm^5^s^2^U_34_, 34 denotes the wobble position) in the wobble base of tRNA [40] (S1 Fig). Therefore, disruption of this pathway appears to promote the persistence of *Salmonella* in macrophages.

### Absence of *yheM* promotes *Salmonella* survival in macrophages in the presence of antibiotics

**Fig 2:**
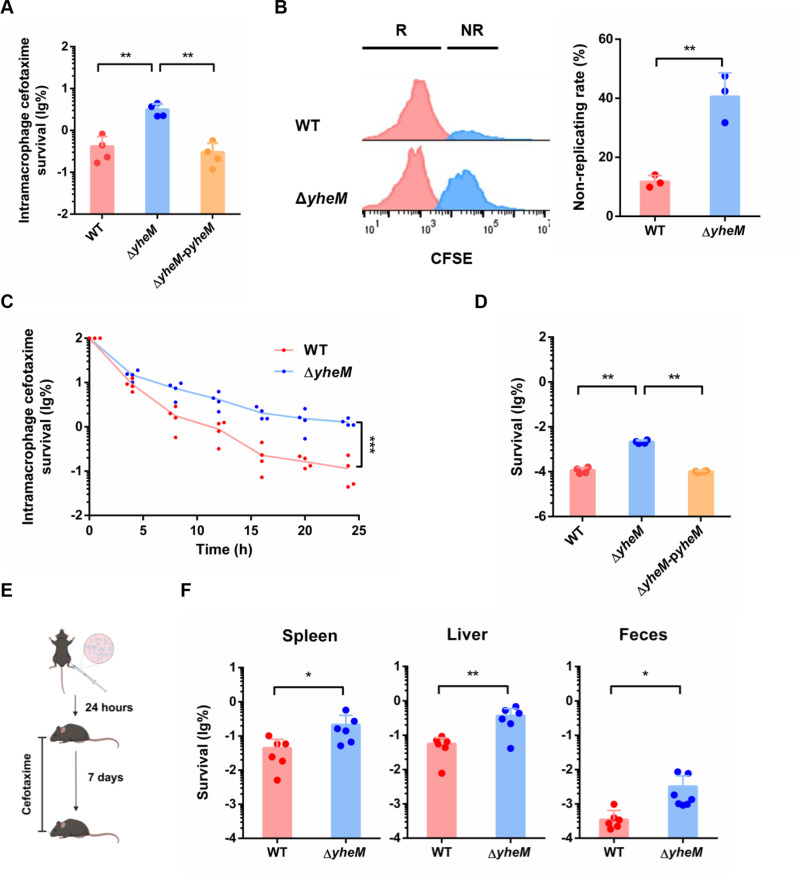
Absence of *yheM* promotes *Salmonella* intramacrophage persistence. (A) Quantification of the percentage of surviving cells for wild-type strain, *yheM* deletion mutant and complementation strain in macrophages in the presence of cefotaxime for 24 hours. (B) Flow cytometry detection of green fluorescence in the wild-type strain and *yheM* deletion mutant labelled with CFSE. Representative fluorescence-activated cell sorting plots (left) of fluorescence dilution experiments tracking the proportion of non-replicating (NR; blue) and replicating (R; red) wild-type strain and *yheM* deletion mutant recovered from intramacrophage after 24 hours of infection and quantification (right). (C) Survival curves of the wild-type strain and *yheM* deletion mutant in macrophages treated with cefotaxime, compared to 2 hours post-infection; lines connect geometric means of each time point for each genotype. (D) Exponential phase cultures of wild-type strain, *yheM* deletion mutant and complementation strain were treated with ampicillin (100 μg/mL) for 5 hours. CFU counts were determined before and after the treatments to calculate the surviving fraction. (E) Schematic of the experimental setup. After 24 hours of intraperitoneal infection with the wild-type strain and *yheM* deletion mutant, mice were treated with cefotaxime for 7 days. Created in BioRender. Tang, H. (2025) https://BioRender.com/q26d345. (F) Percentage of bacteria recovered from the spleen, liver and feces on day 7, and normalized to the average bacterial load at 24 hours post infection. All experiments were performed in triplicate and one representative result is shown. Each point represents the result for one animal. Error bars represent the mean and standard deviation (SD) of four independent samples. *P < 0.05, **P < 0.01 and ***P < 0.001, Student’s *t*-test.

A previous study has shown that the absence of any gene in the thio-modification pathway can effectively block thio-modification activity [41]. To confirm our Tn-seq results, we used the *yheM* deletion mutant to infect mouse macrophage RAW 264.7 cells in combination with cefotaxime treatment. As expected, the *yheM* deletion mutant showed higher intracellular persistence compared to the wild-type strain (Fig 2A). Similarly, we also found that the *yheN* deletion mutant and *trmU* deletion mutant showed higher intracellular persistence compared to the wild-type strain (S2A Fig). However, the *yheM* deletion mutant showed a lower internalization and replication rate in RAW 264.7 cells without antibiotic treatment (S2B Fig). Non-replicating bacterial populations formed after macrophages internalization contribute to the generation of persisters [25]. We then used carboxyfluorescein diacetate succinimidyl ester (CFSE)-labelled bacteria to assess intracellular replication dynamics using flow cytometry. CFSE fluorescence intensity decreases with cell division, so, non-replicating cells retain higher initial fluorescence levels compared to replicating cells [42]. The results showed that deletion of *yheM* dramatically increased the proportion of non-replicating cells within the intracellular population after 24 hours of infection in the absence of antibiotics (Fig 2B). Consistently, the proportion of non-replicating cells in *yheN* deletion mutant and *trmU* deletion mutant was also increased (S2C Fig).

Non-replicating bacterial cells, which exhibit increased survival rates under antibiotic pressure are often associated with antibiotic persistence [25]. We therefore tested whether *yheM* is involved in *Salmonella* persister formation *in vitro*. MIC assays showed that deletion of *yheM* did not affect bacterial susceptibility to cefotaxime and ampicillin (S2D Fig). Interestingly, deletion of *yheM* increased bacterial survival after antibiotics exposure (S2E Fig). We then performed persisters assays in macrophages with cefotaxime treatment. Time-dependent killing curves showed significantly increased survival of the *yheM* deletion mutant in cefotaxime-treated macrophages compared to the wild-type strain, and revealed a biphasic pattern of surviving persisters (Fig 2C). Consistently, deletion of *yheM* showed increased survival of *Salmonella in vitro* treated with cefotaxime or ampicillin (S2F Fig). After 5 hours of treatment, the survival rate of the *yheM* deletion mutant was 10-fold higher than that of the wild-type strain under cefotaxime exposure, and this phenotype could be rescued by complementation (Fig 2D).

*Salmonella* intramacrophage persistence can significantly affect the host response to infection, potentially leading to chronic and recurrent infections. To investigate the role of *yheM* in *Salmonella* antibiotic persistence *in vivo*, we used a mouse model to compare *Salmonella* clearance. We intraperitoneally infected mice with a wild-type strain or a *yheM* deletion mutant. We first assessed bacterial colonization 24 hours post-infection before administering antibiotics. The results showed that deletion of *yheM* resulted in lower bacterial loads in the spleen, liver, and feces (S2G Fig). We then treated infected mice with antibiotics for 6 days and monitored bacterial survival after cessation of the antibiotic treatment (Fig 2E). Consistent with the data from the *in vitro* persister formation assay (Fig 2D), enumeration of bacterial load revealed that the persisters fraction of the *yheM* deletion mutant was much higher than that of the wild-type strain in spleen, liver and feces (Fig 2F). These results suggest that *yheM*-lacking *Salmonella* infected mice experienced prolonged infection and extended shedding.

### Transcriptome analysis of the *yheM* deletion mutant

**Fig 3:**
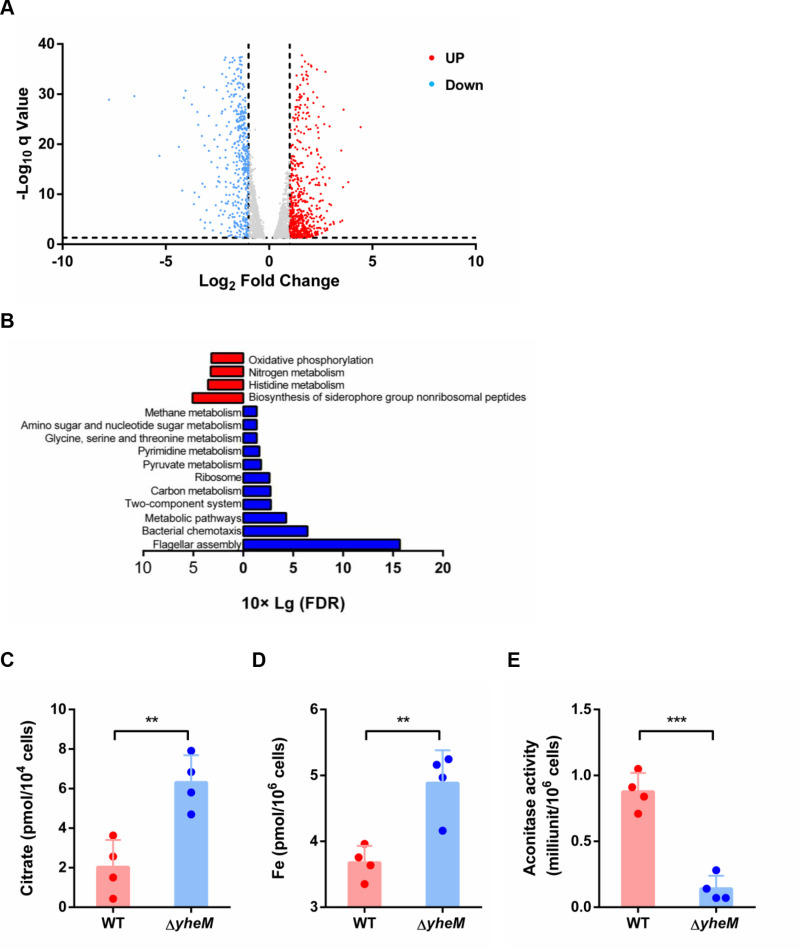
RNA-seq analysis of the *yheM* deletion mutant. (A) Volcano plot showing differentially regulated genes in the *yheM* deletion mutant compared to the wild-type strain. The x-axis is the log_2_ scale of fold change in gene expression, and y-axis is the minus log_10_ scale of q values. Red dots represent significantly upregulated genes with at least 2-fold change, while blue dots represent significantly downregulated genes with at least 2-fold change. (B) Functional KEGG enrichment analysis of differentially expressed genes (DEGs). The vertical axis indicates the name of the differentially expressed gene pathway, and the horizontal axis indicates the 10 log_10_ FDR (false discovery rate). Upregulated enriched pathways are colored red and downregulated enriched pathways are colored blue. (C) Concentration of citrate in the wild-type strain and *yheM* deletion mutant. Exponential phase bacteria were collected by centrifugation and homogenized in ice-cold assay buffer followed by sonication. The amount of citrate was determined by absorbance at 545 nm. (D) Concentration of iron in the wild-type strain and *yheM* deletion mutant. Exponential phase bacteria were collected by centrifugation and homogenized in ice-cold assay buffer followed by sonication. The amount of iron was determined by ICP-MS. (E) Aconitase activity in the wild-type strain and *yheM* deletion mutant. Exponential phase bacteria were collected by centrifugation and homogenized in ice-cold assay buffer followed by sonication. Quantification of aconitase activity was monitored by absorbance at 240 nm. All experiments were performed in triplicate and one representative result is shown. Each point represents an independent sample. Error bars represent the mean and standard deviation (SD) of four independent samples. **P < 0.01 and ***P < 0.001, Student’s *t*-tes*t*.

To explore the mechanism of *yheM-*mediated persistence, we performed RNA sequencing (RNA-seq) in the wild-type strain and the *yheM* deletion mutant. Using criteria of |log_2_ Fold Change| > 2 and a false discovery rate < 0.01, a total of 1284 differentially expressed genes were identified, with 644 genes upregulated and 604 genes downregulated in the *yheM* deletion mutant (Fig 3A). The differentially expressed genes are listed in S2 Dataset. Functional KEGG (Kyoto Encyclopedia of Genes and Genomes) enrichment analysis revealed that these differentially expressed genes were involved in pathways including oxidative phosphorylation, nitrogen metabolism, and various metabolic pathways (Fig 3B). Notably, genes related to citrate metabolism and iron transfer pathway were particularly enriched. In *Salmonella*, citrate metabolism relies on components including the functional citrate transporter CitT, citrate lyase (encoded by *citCDEFXG*) and the two-component regulatory system encoded by *citAB* [43]. Our RNA-seq data showed that deletion of *yheM* strongly suppressed the citrate lyase and citrate transport genes (S3A Fig). Validation by qRT-PCR confirmed these findings, showing a marked decrease in the expression of citrate metabolism-related genes (*citCDEFGTX*) in the *yheM* deletion mutant (S3B Fig).

*Salmonella* requires iron as a cofactor for essential cellular processes involving metalloproteins. To acquire this vital nutrient, *Salmonella* coordinates the activity of an inner membrane protein complex that facilitates the interaction of two TonB-dependent transporters, the ferrichrome transporter (Fhu) and the ferric enterobactin transporter (Fep) [44,45]. RNA-seq data showed that deletion of *yheM* resulted in a significant increase in the transcription of genes associated with the iron uptake and transport regulon (S3C Fig). Based on the decreased expression of citrate metabolism-related genes and the increased expression of iron transport genes, we hypothesized that deletion of *yheM* could lead to accumulation of citrate and iron. To test this hypothesis, we measured citrate and iron concentrations in *Salmonella*. The results showed that the intracellular levels of both citrate (Fig 3C) and iron (Fig 3D) were significantly higher in the *yheM* deletion mutant than in the wild-type strain.

Fe-S cluster proteins are crucial for sensing external signals and maintaining the intracellular redox state of microbial cells. The hemostasis of iron and citrate levels are crucial for Fe-S cluster activity [46]. Therefore, the deletion of *yheM* may affect Fe-S cluster activity. To examine this, we determined the activity of aconitase, which requires 4Fe-4S clusters for its proper function and serves as a marker for Fe-S cluster biogenesis [47,48]. The results showed that the activity of aconitase was significantly reduced in the *yheM* deletion mutant (Fig 3E). These data suggest that deletion of *yheM* disrupted the homeostasis of intracellular citrate and iron metabolism and consequently impaired Fe-S cluster biogenesis.

### Absence of *yheM* promotes persister formation by lowering ATP production via RNS

**Fig 4:**
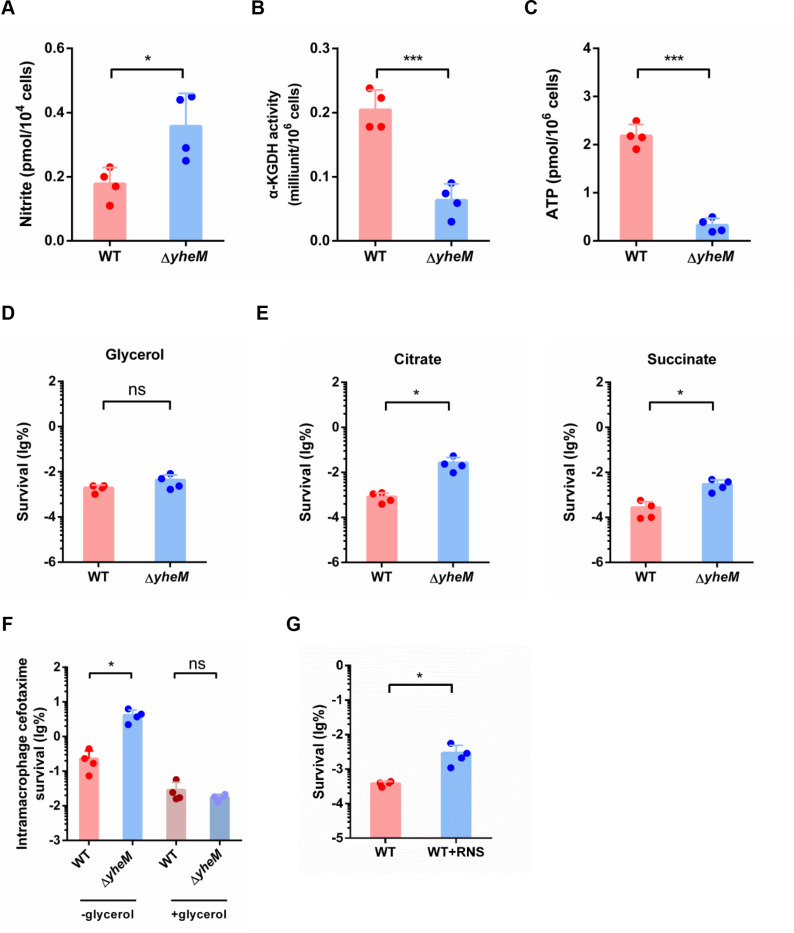
Absence of *yheM* promotes persistence by lowering ATP production *via* RNS. (A) Concentration of nitrite in the wild-type strain and *yheM* deletion mutant in the exponential phase. Bacteria in the exponential phase were collected by centrifugation and homogenized in ice-cold assay buffer followed by sonication. The amount of nitrite was monitored by absorbance at 540 nm. (B) α-KGDH activity in the wild-type strain and *yheM* deletion mutant in the exponential phase. Bacteria in the exponential phase were collected by centrifugation and homogenized in ice-cold assay buffer followed by sonication. Quantification of α-KGDH activity was monitored by absorbance at 340 nm. (C) ATP production in the wild-type strain and *yheM* deletion mutant in the exponential phase. Bacteria in the exponential phase were collected and reagent was added directly to measure luminescence. (D) Cefotaxime survival of the wild-type strain and *yheM* deletion mutant in the exponential phase in the presence of glycerol. Exponential phase cultures of the wild-type strain and mutants were treated with cefotaxime (100 μg/mL) for 5 hours. CFU counts were determined before and after treatment to calculate the surviving fraction. (E) Cefotaxime survival of the wild-type strain and *yheM* deletion mutant in the exponential phase in the presence of citrate or succinate. Exponential phase cultures of the wild-type strain and mutants were treated with cefotaxime (100 μg/mL) for 5 hours. CFU counts were determined before and after treatment to calculate the surviving fraction. (F) Twenty-four hours cefotaxime survival of the wild-type strain and *yheM* deletion mutant in macrophages in DMEM supplemented with 0.2% glycerol normalized to values after 2 hours internalization. (G) Cefotaxime survival of the wild-type strain and *yheM* deletion mutant in the exponential phase in M9 minimal media in the presence of 250 μM Spermine NONOate. All experiments were performed in triplicate and one representative result is shown. Each point represents an independent sample. Error bars represent the mean and standard deviation (SD) of four independent samples. *P < 0.05 and ***P < 0.001, Student’s *t*-tes*t*.

Fe-S cluster proteins exhibit a number of physicochemical properties that support their diverse biological functions, including roles in electron transfer, catalysis, and gene regulation [49]. Dysregulated Fe-S cluster biogenesis can act as sensors to detect redox imbalances caused by reactive oxygen species (ROS) or reactive nitrogen species (RNS) stress. However, no significant differences in ROS were detected between the *yheM* deletion mutant and the wild-type strain (S4A Fig). In contrast, RNS production was much higher in the *yheM* deletion mutant compared to the wild-type strain (Fig 4A). Consistently, RNA-seq analysis showed that the expression of the entire nitrogen metabolism regulon (*nap* cluster and *nar* cluster) was elevated in the *yheM* deletion mutant (S4B Fig). In *Salmonella*, excessive production of RNS disrupts the function of the Fe-S cluster-containing α-ketoglutarate dehydrogenase (αKDH) enzyme complex [50,51]. This complex, composed of SucA, SucB, and LpdA, plays a crucial role in the tricarboxylic acid (TCA) cycle by decarboxylating α-ketoglutarate to produce succinyl-CoA and NADH for ATP production in the respiratory chain [7,52]. Our results showed that deletion of *yheM* caused a significant reduction in αKDH activity (Fig 4B). Impaired αKDH activity can significantly affect ATP production, which prompted us to measure ATP levels. Deletion of *yheM* reduced ATP production by approximately 75% (Fig 4C). Glycerol supplementation in the medium can reduce bacterial persisters in macrophages [7], as glycerol can be utilized as a carbon source in a TCA cycle independent manner. We therefore assessed persister formation in M9CA medium supplemented with either glycerol or citrate or succinate. As expected, the addition of glycerol reduced the differences in persister formation between the mutant and its parental strain in the presence of antibiotic treatment (Fig 4D and 4E). Moreover, glycerol supplementation reduced intramacrophage persister formation of the *yheM* deletion mutant (Fig 4F). As expected, addition of exogenous RNS (NO-releasing compound Spermine NONOate) *in vitro* can enhance *Salmonella* persister formation (Fig 4G). These data support that deletion of *yheM* promotes persister formation by impairing respiratory function and reducing ATP production through increased RNS production.

### (p)ppGpp participates in the persister formation in the stationary phase

**Fig 5:**
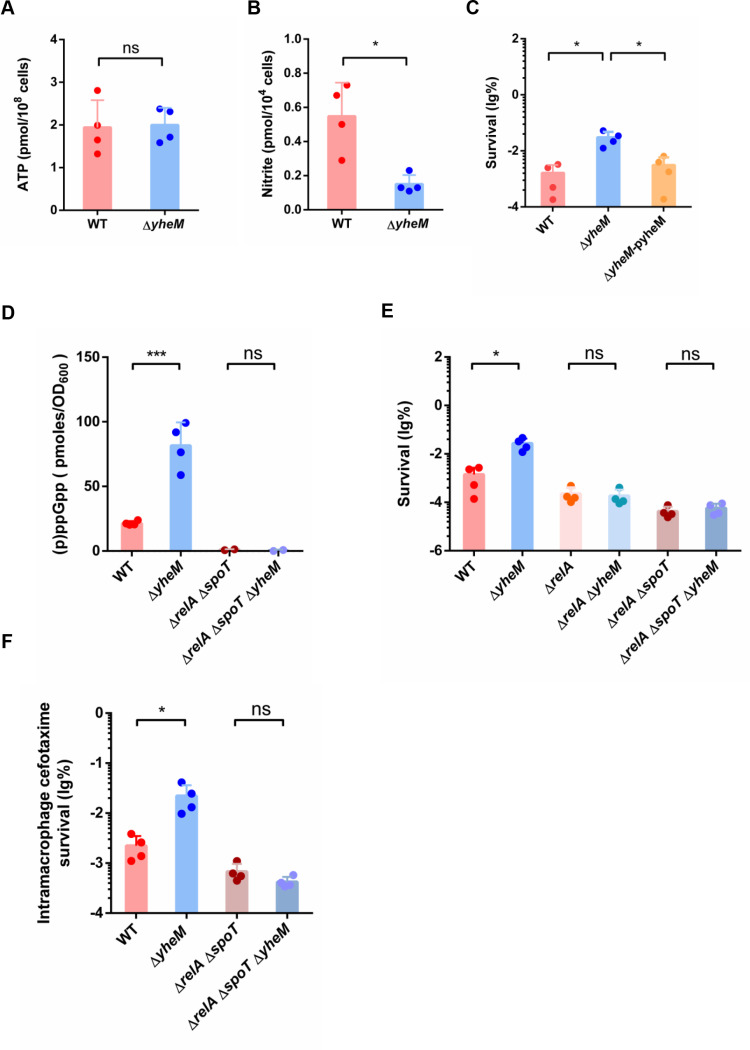
(p)ppGpp participates in the persister formation in the stationary phase. (A) ATP production in the wild-type strain and *yheM* deletion mutant in the stationary phase. Bacteria in the stationary phase were collected and reagent was added directly to measure luminescence. (B) Concentration of nitrite in the wild-type strain and *yheM* deletion mutant in the stationary phase. Stationary phase bacteria were collected by centrifugation and homogenized in ice-cold assay buffer followed by sonication. The amount of nitrite was monitored by absorbance at 540 nm. (C) Stationary phase cultures of the wild-type strain, *yheM* deletion mutant and complementation strain were treated with ampicillin (100 μg/mL) for 5 hours. CFU counts were determined before and after the treatments to calculate the surviving fraction. (D) Concentration of (p)ppGpp in the wild-type strain, *yheM* deletion mutant, *relA* and *spoT* double deletion mutant and *yheM*, *relA* and *spoT* triple deletion mutant. Stationary phase bacteria were collected by centrifugation and homogenized in ice-cold assay buffer for LC-MS/MS analysis. The bacteria were chromatographed using an HPLC system and selective reaction monitoring in negative ion mode with the following transition. (E) Cefotaxime survival of the wild-type strain, *yheM* deletion mutant, *relA* deletion mutant, *relA* and *yheM* double deletion mutant, *relA* and *spoT* double deletion mutant and *yheM*, *relA* and *spoT* triple deletion mutant. Stationary phase cultures of the wild-type strain and mutants were treated with cefotaxime for 5 hours. CFU counts were determined before and after treatments to calculate the surviving fraction. (F) Cefotaxime survival of the wild-type strain, *yheM* deletion mutant, *relA* and *spoT* double deletion mutant and *yheM*, *relA* and *spoT* triple deletion mutant. in macrophages normalized to values after 2 hours internalization. All experiments were performed in triplicate and one representative result is shown. Each point represents an independent sample. Error bars represent the mean and standard deviation (SD) of four independent samples. *P < 0.05 and ***P < 0.001, Student’s *t*-tes*t*.

Given that intracellular ATP is consumed after prolonged growth on limited nutrients, we measured ATP production in *Salmonella* in the stationary phase. The result showed that the *yheM* deletion mutant and the wild-type strain had similar ATP levels (Fig 5A). Our previous data showed that the accumulation of RNS in the *yheM* deletion mutant occurred in the exponential phase (Fig 4A), but this phenotype was completely reversed in the stationary phase. The RNS concentration of the wild-type strain was significantly higher than that of the *yheM* deletion mutant (Fig 5B). Despite the differences in RNS concentration and ATP production between exponential and stationary phase, our *in vitro* persisters assay revealed that the *yheM* deletion mutant still exhibited higher persister formation in the stationary phase than the wild-type strain (Fig 5C). These results suggest that ATP and RNS may not directly mediate persister formation in the stationary phase *yheM* deletion mutant.

In *Saccharomyces cerevisiae*, cells lacking U_34_ 2-thiolation modifications experience slower codon translation rates, leading to the accumulation of aggregated protein [53]. We have previously reported that deletion of *yheM* promotes the aggregation of endogenous protein [39]. In bacteria, codon translation is slowed or stopped in response to amino acid starvation [13]. During amino acid starvation, deacylated tRNAs accumulate and enter the A-site, causing codon translation deceleration [54,55]. RelA, a prototypic member of the RelA/SpoT homologue family, is recruited to stalled ribosomes and activated to synthesize (p)ppGpp, which acts as a pleiotropical secondary messenger [54,56,57]. The (p)ppGpp are the alarmones in the stringent response that regulate multiple cellular processes, and are known to be associated with persister formation [58,59]. We hypothesized that the slower codon translation resulting from the absence of *yheM* would facilitate the activation of RelA and (p)ppGpp production. To test this hypothesis, we determined (p)ppGpp production in mutant and wild-type strains in the stationary phase. As expected, the *yheM* deletion mutant exhibited 3-fold higher levels of (p)ppGpp compared to the wild-type strain (Fig 5D). Deletion of *relA* and *spoT* completely blocked (p)ppGpp production. These results support that deletion of *yheM* activates (p)ppGpp synthesis.

Next, we investigated whether (p)ppGpp affects antibiotic persistence in the *yheM* deletion mutant. To this end, we performed a persistence assay with the *yheM* deletion mutant and the wild-type strain with ampicillin in the stationary phase. Consistently, deletion of *relA* alone or both *relA* and *spoT* in the *yheM* deletion strain reduced persister formation (Fig 5E). Correspondingly, deletion of *relA* and *spoT* in the *yheM*-deficient strain reduced the proportion of intramacrophage persisters to the same level as in the wild-type strain (Fig 5F). Furthermore, deletion of *relA* and *spoT* did not prevent persister formation in exponential phase *yheM* deletion mutant (S5A Fig), possibly because these strains could not accumulate sufficient (p)ppGpp to respond to antibiotic treatment in the exponential phase. As endogenous protein aggregation is an important indicator of the depth of bacterial dormancy [17], we examined endogenous protein aggregation in the mutants. We found that deletion of *yheM* promoted endogenous protein aggregation, whereas *relA* and *spoT* were dispensable for endogenous protein aggregation (S5B Fig).

### Sulfur transfer system is involved in *Salmonella* multidrug persistence

**Fig 6:**
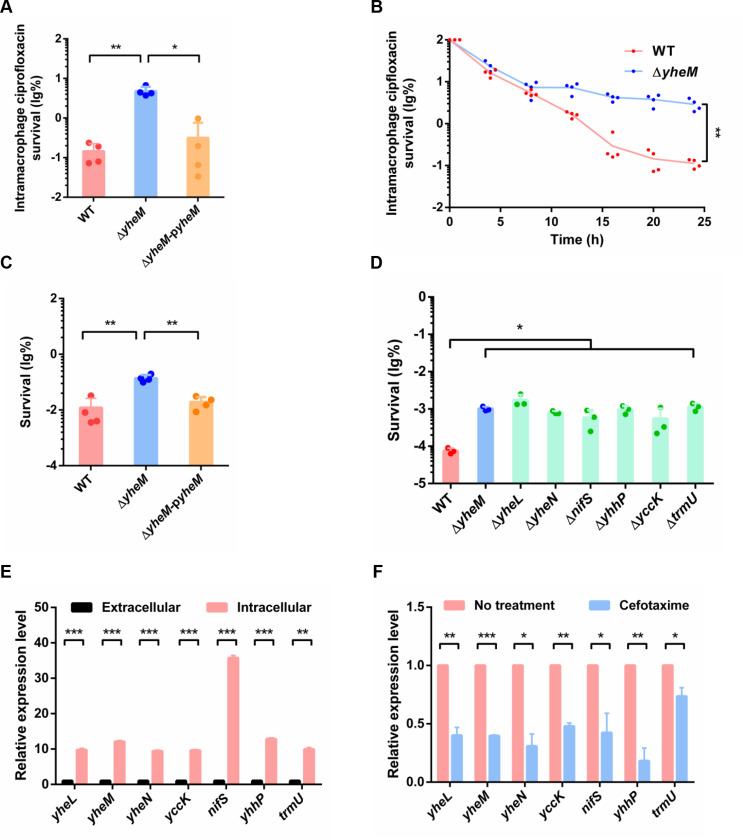
Sulfur transfer system is involved in multidrug persistence. (A) Ciprofloxacin survival of the wild-type strain, *yheM* deletion mutant and complementation strain in macrophages at 24 hours normalized to values after 2 hours internalization. (B) Survival curves of the wild-type strain and *yheM* deletion mutant in macrophages treated with ciprofloxacin, compared to 2 hours post-infection; lines connect geometric means of each time point for each genotype. (C) Exponential phase cultures of the wild-type strain, *yheM* deletion mutant and complementation strain were treated with ciprofloxacin for 5 hours. CFU counts were determined before and after treatment to calculate the surviving fraction. (D) Cefotaxime survival of the wild-type strain and each sulfur transferase gene deletion mutant. Exponential phase cultures of the wild-type strain and mutants were treated with cefotaxime for 5 hours. CFU counts were determined before and after treatments to calculate the surviving fraction. (E) Relative expression level of sulfur transferase genes in macrophages compared to expression after 24 hours of cefotaxime treatment, determined by qRT-PCR. All experiments were performed in triplicate and one representative result is shown. Each point represents an independent sample. Error bars represent the mean and standard deviation (SD) of four independent samples. *P < 0.05, **P < 0.01 and ***P < 0.001, Student’s *t*-test. (F) Relative expression level of sulfur transferase genes in macrophages compared to in vitro expression determined by qRT-PCR. Error bars represent mean and standard deviation (SD). **P < 0.01; ***P < 0.001, Student’s *t*-test.

Given that persisters exhibit a multidrug tolerance phenotype [60], we speculated that *yheM* may mediate multidrug persistence in *Salmonella*. Persisters assays were also performed with ciprofloxacin (quinolones), doxycycline (tetracyclines) or kanamycin (aminoglycosides) at 100 × MIC for 5 hours. Time-dependent killing curves showed significantly increased survival of the *yheM* deletion mutant to antibiotic treatment compared to the wild-type strain, and revealed a biphasic pattern of surviving persisters (S6A Fig). MIC assays showed that deletion of *yheM* did not affect bacterial susceptibility to multiple antibiotics (S6B Fig). In addition, a survival assay in macrophages with ciprofloxacin showed that the *yheM* deletion mutant generated more intramacrophage persisters compared to the wild-type strain (Fig 6A and 6B). After 5 hours of antibiotic treatment, the *yheM* deletion mutant exhibited significantly higher persister formation than the wild-type strain, and this phenotype could be rescued by complementation (Fig 6C). These results indicate that deletion of *yheM* promotes persister formation in *Salmonella* during exposure to multiple antibiotics. We then explored the role of other sulfur transferase components in persister formation. Consistent with the above observations, deletion of any genes involved in the tRNA s^2^U_34_ thio-modification pathway promoted persister formation (Fig 6D). This finding suggests that low levels of tRNA s^2^U modification cause high persister formation in *Salmonella*. To test this hypothesis, we determined the transcription levels of sulfur transferase genes in *Salmonella* during macrophage infection and antibiotic treatment. Our qRT-PCR analysis revealed an interesting phenomenon of increased sulfur transferase gene expression within macrophages (Fig 6E) and a significant decrease in transcription after antibiotic treatment (Fig 6F).

## Discussion

**Fig 7:**
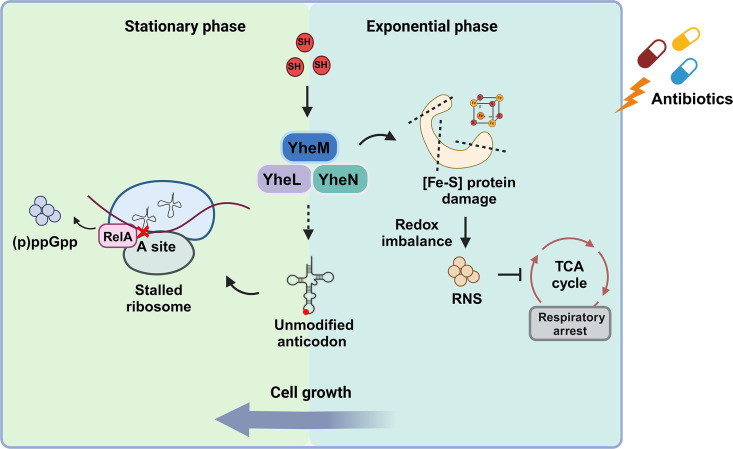
The model of this work. The *yheM* deletion mutant uses two different mechanisms to respond to antibiotic stress at different growth phases. During the log phase, the deletion of *yheM* leads to disruption of iron-sulfur clusters and the redox balance, causing an increase in RNS levels. This accumulation of RNS inhibits bacterial respiration and ATP generation. In contrast, during the stationary phase, as the growth rate decreases, the effect of RNS on growth is no longer dominant. Instead, the reduced translation rate due to the absence of *yheM* activates RelA, leading to the accumulation of (p)ppGpp in the cells. Created in BioRender. Tang, H. (2025) https://BioRender.com/n93r706.

Persister cells in bacterial infections contribute to their chronicity and are difficult to eradicate with conventional antibiotics. Examples include tuberculosis, typhoid fever, Lyme disease, and recurrent urinary tract infections [29,61–63]. Recent studies across various pathogens have provided valuable insights into how antibiotic persistence is initiated when bacteria encounter their host environment [64–66]. However, there remains a significant gap in our knowledge of the specific molecular factors that promote persister formation during infection. In this study, we used the *Salmonella* transposon mutation library to identify key genes that influence *Salmonella* antibiotic persistence in macrophages. Our results showed that the sulfur transferase in the tRNA thio-modification pathway contributes to *Salmonella* persistence through different mechanisms in different phases (Fig 7).

### The bottleneck of our Tn-seq experiment

The bottleneck effect of Tn-seq is typically caused by certain stochastic processes that lead to a drastic reduction in population size [67]. In our cellular infection model, the bottleneck effect may occur during the process of the transposon mutant library entering macrophages. To rule out this bottleneck effect, we first determined the number of the intracellular bacteria after 2 hours infection (input). CFU counting revealed that the input bacterial number was 1,475,000. Our transposon mutant library contains approximately 70,000 distinct insertions. Therefore, the number of input bacteria is 20 times the library capacity. Secondly, comparisons between the original mutant library and the input library revealed a high degree of correlation (R^2^ = 0.9482), indicating that the process of infection did not result in the loss of mutant abundance. Both results suggest that our Tn-seq screening has minimal bottleneck effects.

### Novel role of tRNA thio-modification in persister formation

The mechanisms underlying bacterial persister formation are diverse, and several studies have highlighted the role of tRNA modifications in this process. For example, TacT, an acetyltransferase, blocks the formation of peptide bonds between nascent peptides and tRNA by modifying the primary amine group of amino acids on tRNA, and this inhibition of protein translation promotes persister formation [14]. In addition, methylation of the G_37_ site on tRNA m1 in Gram-negative bacteria contributes to the maintenance of a double membrane structure associated with drug resistance and persistence [68]. Our work showed that the *yheM* deletion strain, lacking s^2^U_34_ modifications, experienced protein aggregation leading to RelA activation, (p)ppGpp production and persister formation in the stationary phase. Furthermore, deletion of genes involved in the tRNA s^2^U_34_ thio-modification pathway, including *yheLMN*, *nifS*, *yccK*, *trmU*, *yhhP*, also promoted persister formation. Taken together, our data indicated that tRNA thio-modification plays an important role in persister formation.

### The sulfur transferase mediates persistence in different growth phases

Our study suggests that RNS restricts the *yheM* deletion mutant from generating enough energy through the TCA cycle to support bacterial growth, leading to a decrease in ATP levels in the exponential stages, which in turn increases persistence. Even when bacteria experience nutrient deprivation within the host, our work indicates that RNS still influences bacterial persistence. Consistently, exogenous RNS can significantly enhance *Salmonella* persister formation. Previous studies have pointed out that during infection, nitrosative stress induces stronger resistance to doxycycline treatment in *Yersinia pseudotuberculosis* in host tissues, thereby allowing more survival [69], suggesting that bacterial RNS stress alone was sufficient to alter antibiotic susceptibility. Host-derived RNS also enhances bacterial persistence during infection, and reducing host-derived RNS can restore sensitivity of *Salmonella* to antibiotics in macrophages [7]. Overall, these studies support the broad impact of RNS on antibiotic persistence and suggest that the production of RNS weakens the effect of antibiotics, making it a key factor in the formation of recurrent infections.

However, we also noticed that the differences in RNS between the *yheM* deletion mutant and WT are not constant, and the reversal of this difference in the stationary phase prompted us to explore a persistence mechanism suitable for the stationary phase. Several studies have shown that stationary phase bacterial cultures typically have higher proportions of persisters compared to exponentially growing populations [11,70,71]. Changes in energy metabolism and respiratory chain activity can alter bacterial persistence levels. (p)ppGpp is considered to be a stationary phase or growth arrest-specific messenger, and during the exponential phase, (p)ppGpp is produced at low basal level and the effect of (p)ppGpp on global gene expression in (p)ppGpp-deficient mutants is similar to that in the wild-type strain [72]. In this study, we found that defects in (p)ppGpp synthesis do not affect the persistence of the *yheM* deletion mutant in the exponential phase. Furthermore, RNA-seq analysis revealed that genes related to citrate metabolism and iron transfer pathways were particularly enriched in the *yheM* deletion strain in the exponential phase. In this phase, reduced ATP production, caused by elevated RNS production, may contribute to persister formation. As bacterial cell growth and nutrient availability decrease, intracellular ATP levels decline while (p)ppGpp accumulates. At this point, the regulatory role of (p)ppGpp on gene expression becomes the predominant factor in persister formation. Thus, *yheM* deletion enhances (p)ppGpp synthesis in the stationary phase, thereby promoting persister formation. Additionally, a study has suggested that in minimal medium, exogenous RNS stimulation can also induce (p)ppGpp production in *Salmonella* through the creation of branched-chain amino acid nutritional deficiencies in a RelA-dependent manner [73]. In our study, we found that deletion of *relA* alone or both *relA* and *spoT* did not prevent persister formation in the exponential phase in *yheM* deletion mutant. This indicated that (p)ppGpp does not affect *Salmonella* persistence during the exponential phase. We hypothesized that these strains could not accumulate sufficient (p)ppGpp to respond to antibiotic treatment in the exponential phase. In the stationary phase, RNS levels in the *yheM* deletion strain decreases and no longer plays a major role in persister formation. At this stage, due to branched-chain amino acid nutritional deficiency or the loss of thio-modifications, ribosomal stalling occurs and stimulates RelA to synthesize (p)ppGpp, thereby affecting *Salmonella* persistence. Therefore, these data highlight that metabolic reprograming caused by the sulfur transferase deficiency drive increased persistence through different mechanisms in different phases (Fig 7).

### Intracellular persisters and chronic persistent infections

Some pathogenic bacteria, such as *Salmonella*, not only survive within macrophages but also replicate within them. Once internalized by macrophages, bacterial stress responses drive the formation of intracellular persisters [25]. In this study, we show that sulfur transferase genes promote *Salmonella* persistence in macrophages. Additionally, the proportion of persisters in the *yheM* deletion mutant was significantly higher than in the wild-type strain in mice. These results suggest that *Salmonella* lacking sulfur transferase experience persistent infection and enhanced antibiotic resistance. Our previous data show that the absence of the sulfur transferase impairs *Salmonella* virulence and adaptation to various environmental stresses. Therefore, this diversity in virulence and persistence underpins an important aspect of bacterial heterogeneity during infection.

*Salmonella* are exposed to multiple stressors within macrophages, including nutrient deprivation, reactive oxygen species, and reactive nitrogen species. Adaptation to these stressors has a profound impact on the transcriptome of *S*. Typhimurium. Our qRT-PCR results show an increase in the expression of sulfur transferase genes in macrophages, suggesting that these sulfur transferase genes are essential for *Salmonella* replication in macrophages. Consistent with this observation, deletion of *yheM* inhibited *Salmonella* replication in macrophages. Interestingly, the expression of these genes was reduced after antibiotic treatment. It appears that intramacrophage *Salmonella* may promote persister formation by downregulating the expression of these sulfur transferase genes. Therefore, we propose that in order to adapt to intramacrophage stress, *Salmonella* could enhance the expression of sulfur transferase genes to achieve intramacrophage survival and replication. Once encountering antibiotics exposure during infection, *Salmonella* could decrease these sulfur transferase genes expression to form more persisters to survive antibiotic treatment. Admittedly, the gene transcriptional regulatory mechanism involved in this process remains unknown and is worth investigating in the future. In conclusion, this study provides new insights into the regulatory mechanisms that enable *Salmonella* to balance virulence and persistence, thereby facilitating persistent infection in mammalian cells.

## Materials and methods

### Ethics statement

All animal procedures were approved by Shanghai Jiao Tong University School of Medicine, and this study was carried out in strict accordance with the National Research Council Guide for Care and Use of Laboratory Animals [SYXK (Shanghai 2018–0027)]. All surgery was performed under sodium pentobarbital anesthesia, and all efforts were made to minimize suffering.

### Bacterial strains and plasmids construction and culture conditions

The bacterial strains and plasmids used in this study are listed in S1 Table. The mutant was constructed by deleting and replacing the gene with a cassette encoding chloramphenicol resistance. A fragment carrying the resistance gene obtained by polymerase chain reaction from plasmid pKD3 was then transformed to *Salmonella* Typhimurium strain 14028s harboring the λ red expression plasmid pKD46. All mutants were verified by PCR and sequencing. To construct the *yheM* expression plasmid, *yheM* was amplified from *Salmonella* and cloned into pBAD33. The sequence of the expression plasmids was confirmed by sequencing. All primers used in this study are listed in S2 Table. *Salmonella* Typhimurium strain 14028s and mutant strains were grown routinely at 37°C in Lysogeny Broth (LB) or M9CA minimal medium. The antibiotics used were 100 μg/mL ampicillin and 17 μg/mL chloramphenicol. All strains were stored at -80°C in LB medium containing 15% glycerol.

### *In vitro* selection of the transposon mutant library

The *Salmonella* transposon mutant library used in this study was previously constructed and stored in aliquots at -80°C. Prior to screening, an aliquot of the transposon libraries was taken from storage at -80°C, thawed at room temperature and diluted 1:10 in LB. The library was recovered at 37°C with shaking at 250 rpm for 1 hour. The recovered library contained 5 × 10^9^ CFUs of mutants per mL. RAW 264.7 cells were maintained in Dulbecco’s modified Eagle’s medium supplemented with 10% (v/v) fetal bovine serum, and used for infection at passage 10 or less. Transposon library cultures grown overnight in LB containing 17 μg/mL chloramphenicol were opsonized in 15% normal mouse serum and PBS at 37°C for 15 minutes. Bacteria were pelleted, resuspended in PBS and used to infect 5 × 10^6^ macrophage cells at an MOI of 100 for 2 hours. The cells were rinsed in PBS, treated with Dulbecco’s modified Eagle’s medium supplemented with 10% (v/v) fetal bovine serum and 100 μg/mL gentamicin for 1 hour, then one third of the intracellular bacteria were harvested and the others were maintained in 100 μg/mL cefotaxime and 25 μg/mL gentamicin for the duration of the infection. After 24 hours, macrophages were lysed with 1% Triton X-100 and all bacteria were harvested for genomic DNA extraction. A total of three independent biological replicates, each containing approximately 70,000 individual insertion mutants, were used to ensure robust coverage of the library.

### Antibiotic survival assays in infected macrophages

Bacteria were harvested and washed three times with PBS, 1 × 10^9^ cells are added to an equal volume of CFSE at 10 μM and incubated at 37°C for 15 minutes with agitation. The labelling reaction is stopped for 10 minutes by adding an equal volume of heat inactivated fetal bovine serum. The CFSE labelled bacterial cells are washed twice with PBS and used to infect 2 × 10^5^ macrophage cells at an MOI of 50 for 2 hours. The cells were rinsed in PBS, treated with Dulbecco’s modified Eagle’s medium supplemented with 10% (v/v) fetal bovine serum and 100 μg/mL gentamicin for 1 hour, then maintained in 100 μg/mL cefotaxime and 25 μg/mL gentamicin for the duration of infection. After 24 hours, the macrophages were lysed with 1% Triton X-100 and the bacteria were harvested and analyzed on a flow cytometer (Beckman), then serially diluted tenfold in PBS and drops of 200 μL were plated on LB agar to determine the number of CFU. For the survival curve, bacteria were harvested after 4 hours, 8 hours, 12 hours, 16 hours, 20 hours and 48 hours of antibiotic treatment. Ten-fold serial dilutions were made in PBS and bacteria were plated on LB agar for CFU enumeration.

### *In vivo* antibiotic survival assay

Bacterial cultures were diluted 1:50 in fresh LB and incubated on a shaker (200 rpm) at 37°C for 1.5 hours for exponential phase and 10 hours for stationary phase. For the experiment with different carbon sources, 10 μL of the overnight culture were diluted in 500 μL of M9CA minimal medium containing 0.2% glycerol, succinate, or citrate as the sole carbon source. For the exogenous RNS treatment experiment, 10 μL of the overnight culture were diluted in 500 μL of M9CA minimal medium containing 0.2% glucose. After 30 minutes of incubation at 37°C, the medium was supplemented with 0 or 250 μM Spermine NONOate. The cultures were then exposed to different antibiotics including 100 μg/mL cefotaxime, 100 μg/mL ampicillin, 100 μg/mL ciprofloxacin, 150 μg/mL kanamycin, 200 μg/mL doxycycline and 200 μg/mL ampicillin for 5 hours. Initial cell counts were determined by taking 10 μL and serially diluting in phosphate-buffered saline (PBS), washing and plating on LB agar. CFU counts were measured after overnight incubation at 37°C.

### Antibiotic survival assays in infected mice

The *yheM* deletion mutant and wild-type strains used for all mouse experiments were grown in LB overnight for 16 hours prior to infection. Eight-week-old SPF C57BL/6 mice were housed in cages of five. Four cages of C57BL/6 were inoculated intraperitoneally with 200 μL of PBS containing approximately 1 × 10^8^ CFU of *yheM* deletion mutant or wild-type strain. Two cages of mice were sacrificed 24 hours after infection. Mice were killed by carbon monoxide asphyxiation followed by cervical dislocation. The other cages were treated with cefotaxime (150 mg/kg orally every 12 hours). After 6 days of treatment, the mice were sacrificed. Spleen, liver and feces were collected from each mouse and mechanically homogenized in cold PBS. Samples were diluted in PBS and 10-fold serial dilutions were plated on LB agar (200 μL drops). Plates were incubated at 37°C for 24 hours to assess the bacterial load in the mice.

### RNA sequencing

Two independent cultures of the *yheM* deletion mutant and wild-type strain were grown in LB broth to late exponential phase (OD_600_ = 1.0) by subculturing (1:100) overnight cultures into fresh LB for 2.5 hours at 37°C. Total RNA was isolated from bacterial cultures using the RNAprep Pure Cell/Bacteria Kit (Tiangen, Beijing, China). RNA samples were quantified and reverse transcribed using the random hexamers. Quantitative real-time PCR (qRT-PCR) was performed using ChamQ SYBR Color qPCR Master Mix (Vazyme) in a 20 µL reaction mixture. Fold changes in gene expression were calculated using the 2^-ΔΔ^Ct method.

One microgram of total RNA was used for library preparation and loaded onto an Illumina HiSeq 2000 instrument for sequencing. Cutadapt was used to process the technical sequences in fastq format. Clean data were aligned to the reference genome using Hisat2 software (v2.0.1). Differential expression analysis was performed using the DESeq2 Bioconductor package, and Padj of genes was set < 0.05 to detect differentially expressed genes.

### Measurement of cellular ATP

Bacterial cultures were diluted 1:50 in fresh LB and samples were collected after 1.5 hours for exponential phase and 10 hours for stationary phase at 37°C. The ATP content of each sample was measured using the BacTiter Glo Kit (Promega) according to the manufacturer’s instructions. Luminescence was recorded using a microplate luminometer.

### Quantification of nitrite

Nitrite quantification was performed using a Nitrite Content Assay Kit (BoxBio). Briefly, bacterial cultures were diluted 1:50 in fresh LB and samples were collected after 1.5 hours for exponential phase and 10 hours for stationary phase at 37°C. Samples were collected by centrifugation and homogenized in 100 µL of ice-cold assay buffer followed by sonication. The supernatant (65 µL) was collected in a fresh tube and used for subsequent steps according to the manufacturer’s protocol. Samples have a characteristic absorption peak at 540 nm and can be detected using a Multiskan SkyHigh Microplate Spectrophotometer (Thermo).

### Quantification of ROS

Bacterial cultures were diluted 1:50 in fresh LB and samples were collected after 1.5 hours for exponential phase. ROS generation was determined using a ROS assay kit (Beyotime) according to the manufacturer’s protocol. The fluorescence signal of ROS was quantified at 488/525 nm (excitation/emission).

### Quantification of iron

Bacterial cultures were diluted 1:50 in fresh LB and samples were collected after 1.5 hours for exponential phase. Cells were collected by centrifugation at 5,500 rpm for 10 minutes at room temperature, resuspended in 2 mM EDTA and washed by centrifugation at 5,500 rpm for 10 minutes, three times. Resuspend in 10 mL ddH_2_O and aliquot into 2 mL screw-capped tubes. Disrupt the cells using a cell disruptor by vortexing 10 times for 1 minute each with 1 minute intervals on ice. Centrifuge at 5,000g for 10 minutes at 4°C to remove unbroken cells. Carefully transfer the supernatant, filter through a 0.22 μm filter and send for analysis. Samples were analyzed by ICP-MS at the Instrumental Analysis Centre, Shanghai Jiao Tong University.

### Aconitase assay

Aconitase assays were performed using an Aconitase (ACO) Activity Assay Kit (BoxBio). Briefly, bacterial cultures were diluted 1:50 in fresh LB and samples were collected after 1.5 hours at 37°C. Samples were collected by centrifugation and homogenized in 100 µL of ice-cold assay buffer followed by sonication. Intact cells and cell debris were removed by centrifugation at 2,000g for 15 minutes at 4°C. The supernatant (65 µL) was collected in a fresh tube and used for subsequent steps according to the manufacturer’s protocol. The samples have a characteristic absorption peak at 240 nm and can be detected using a Multiskan SkyHigh Microplate Spectrophotometer (Thermo).

### Citrate assay

Citrate assays were performed using a citric acid (CA) content assay kit (BoxBio). Briefly, bacterial cultures were diluted 1:50 in fresh LB and samples were collected after 1.5 hours at 37°C. Samples were collected by centrifugation and homogenized in 100 µL of ice-cold assay buffer followed by sonication. Intact cells and cell debris were removed by centrifugation at 2,000g for 15 minutes at 4°C. The supernatant (65 µL) was collected in a fresh tube and used for subsequent steps according to the manufacturer’s protocol. The samples have a characteristic absorption at 545 nm and can be detected using a Multiskan SkyHigh microplate spectrophotometer (Thermo).

### HPLC-based quantification of (p)ppGpp

Harvest cells by placing 10–40 mL of culture on ice-cold formic acid (1 M final) and rapidly freeze in liquid nitrogen. Quickly thaw in 37°C water bath and 30 minutes on ice with occasional vortexing. Centrifuge at 5,000 g (10 minutes, 4°C) and decant the supernatant directly into a syringe with a 0.2 µm filter. Apply the diluted sample at 20x on a Q-Sepharose FF column (~6.5 mL/min). Wash with cold mQ (2–3 minutes, ~ 6.5 mL/min) and elute at 1 mL/min with 2 M LiCl, 25 mM Tris pH 8. Transfer the samples to -20°C for O/N precipitation or precipitate on ice for 10 minutes and centrifuge at 5,525 G (20 minutes, 4°C) on a swing-out rotor. Wash the pellet with 70% EtOH and centrifuge again. Dry the pellet by freeze-drying (short freeze-drying is sufficient, about 20 minutes) and resuspend the dried pellet in cold water (200 µL). Transfer the samples to cold 1.5 mL microcentrifuge tubes and centrifuge for at least 30 minutes at maximum speed, 4°C. Collect the supernatant in a fresh tube for HPLC analysis.

Aliquot 300 μL of sample into a clean 1.5 mL tube, vortex at 800 g for 10 minutes at 4°C, then centrifuge (4°C, 9,600 g) for 10 minutes. Transfer 200 μL of the supernatant to another clean 1.5 mL tube and dry the supernatant on nitrogen. Add 300 μL of cold reconstituted solution (water/methanol, v/v,1/4), mix well and collect for LC-MS/MS analysis. Two microliters of solution were chromatographed on an Agilent 1290 UHPLC system equipped with a 4.6*100 mm, 3.5 μM XBridge Amide column (Waters, Milford, MA) at a flow rate of 0.4 mL/min. Solvent A (10 mM ammonium acetate and 0.2% ammonium hydroxide in water) was held at 95% for 0.8 minutes, followed by a linear gradient to 95% solvent B (10 mM ammonium acetate and 0.2% ammonium hydroxide in acetonitrile) for the next 4.6 minutes. The column was held at 95% B for 1 minute and then equilibrated to 95% A for 3.6 minutes. The needle was washed with a mixed solvent (50% methanol in water) before each injection. Selective Reaction Monitoring (SRM) was performed in negative ion mode using an AB SCIEX 6500 plus QTRAP with the following transition: m/z 602.0/ 503.8 for (p)ppGpp.

### Statistical analyses and data quantification

Data used for quantification were obtained from at least three biological replicates, and GraphPad Prism 8 (GraphPad Software) was used for statistical analysis and quantification. Student’s *t*-tests (two-tailed) were used to compare two groups, whereas analysis of variance (ANOVA) was used to compare more than two groups. P values less than 0.05 were considered significant (*P < 0.05, **P < 0.01 and ***P < 0.001), and values of more than 0.05 were considered not significant.

## Supporting information

S1 Fig The sulfur-relay system for s^2^U_34_ modification. Created in BioRender. Tang, H. (2025) https://BioRender.com/u23k795.
(TIF)

S2 Fig Persistence test of the *yheM* deletion mutant. (A) Quantification of the percentage of surviving cells for wild-type strain and *yheN* deletion mutant and *trmU* deletion mutant in macrophages in the presence of cefotaxime for 24 hours. (B) Effects of *yheM* on *Salmonella* internalization (left) and replication (right) in macrophages. Macrophages were infected with wild-type strain or *yheM* deletion mutants. (C) Flow cytometry detection of green fluorescence in the wild-type strain and *yheN* deletion mutant and *trmU* deletion mutant labelled with CFSE. Representative fluorescence-activated cell sorting plots. (D) MIC tests of the wild-type strain and *yheM* deletion mutant with cefotaxime (Cef) and ampicillin (Amp). (E) Survival of the wild-type strain and *yheM* deletion mutant after exposure to 2 × MIC of cefotaxime (Cef) and ampicillin (Amp) for 2 hours. (F) Time-dependent killing curves of the wild-type strain and *yheM* deletion mutant in the exponential phase treated with ampicillin (left) or cefotaxime (right). (G) Bacterial load in tissues 24 hours after infection. Livers and spleens were harvested and the number of bacteria counted. Feces were collected and quantified at 24 hours post-infection. All experiments were performed in triplicate and one representative result is shown. Each point represents the result for one animal. Error bars represent the mean and standard deviation (SD) of four independent samples. *P < 0.05, **P < 0.01 and ***P < 0.001, Student’s *t*-test.
(TIF)

S3 Fig Absence of *yheM* impairs Fe-S cluster biogenesis. (A) Heat map based on RNA-seq results showing the relative transcription of genes involved in the citrate lyase and citrate transport regulon between the wild-type strain and the *yheM* deletion mutant. Cluster analysis was performed using the log_10_(FPKM+1) values and standardized by Z-score. (B) The relative expression level of the citrate regulon in the *yheM* deletion mutant (blue) compared to the expression in the wild-type strain (red) as determined by qRT-PCR. RNA was extracted from exponential phase *Salmonella* cultures. (C) Heat map based on RNA-seq results showing the relative transcription of genes involved in the iron transfer system between the wild-type strain and the *yheM* deletion mutant. Cluster analysis was performed using the log_10_(FPKM+1) values and standardized by Z-score.
(TIF)

S4 Fig Absence of tRNA thio-modification promoted RNS production. (A) ROS production in the wild-type strain and *yheM* deletion mutant in the exponential phase. The fluorescence signal of ROS was quantified at 488/525 nm (excitation/emission) (B) Heat map based on RNA-seq results showing the relative transcription of genes involved in the nitrogen metabolism regulon between the wild-type strain and the *yheM* deletion mutant. Cluster analysis was performed using the log_10_(FPKM+1) values and standardized by Z-score.
(TIF)

S5 Fig Exponential phase persister formation of the *yheM* deletion mutant is independent of (p)ppGpp. (A) Cefotaxime survival of the wild-type strain, *yheM* deletion mutant, *relA* deletion mutant, *relA* and *yheM* deletion mutant, *relA* and *spoT* deletion mutant and *yheM*, *relA* and *spoT* triple deletion mutant in the exponential phase. All experiments were performed in triplicate and one representative result is shown. Each point represents an independent sample. Error bars represent mean and standard deviation (SD). **P < 0.01; ***P < 0.001, Student’s *t*-test. (B) Protein aggregates isolated from the wild-type strain, *yheM* deletion mutant, *relA* and *spoT* double deletion mutant and *yheM*, *relA* and *spoT* triple deletion mutant in macrophages at 37°C. Total cell lysates were used to indicate identical protein concentrations between samples.
(TIF)

S6 Fig Sulfur transfer system is involved in *Salmonella* multidrug persistence. (A) Time-dependent killing curves of the wild-type strain and *yheM* deletion mutant treated with ciprofloxacin (200 μg/mL), doxycycline (200 μg/mL) and kanamycin (100 μg/mL). (B) MIC determination of the wild-type strain and *yheM* deletion mutant with ciprofloxacin (Cip), doxycycline (Dox), tetracycline (Tet), gentamicin (Gen), kanamycin (Kan) and polymyxin B (PMB).
(TIF)

S1 Table Bacterial strains and plasmids used in this study. (DOCX)

S2 Table Primers used in this study. (DOCX)

S1 Dataset Tn-seq analysis. (XLSX)

S2 Dataset RNA-seq analysis of the *yheM* deletion mutant. (XLSX)
