# Correction: Disruption of sulfur transferase complex increases bacterial intramacrophage persistence

**DOI:** 10.1371/journal.ppat.1014064

**Published:** 2026-03-19

**Authors:** Huang Tang, Zuoqiang Wang, Congcong Li, Jingchen Yu, Wanqiu Huang, Tao Zhou, Chuanzhen Zhang, Bingjie Wen, Chengyue Wang, Xiaocen Zhu, Danni Wang, Jing Tao, Jie Lu, Jinjing Ni, Yu-Feng Yao

The raw data underlying Figs 2-6 and S2-S6 were not originally provided with this article [1]. The authors have provided the data as S1–S10 Files.

With this Correction, all relevant data are now provided in the Supporting Information files.

## Supporting information

S1 File Raw data underlying Fig 2. (XLSX)

S2 File Raw data underlying Fig 3. (XLSX)

S3 File Raw data underlying Fig 4. (XLSX)

S4 File Raw data underlying Fig 5. (XLSX)

S5 File Raw data underlying Fig 6. (XLSX)

S6 File Raw data underlying Fig S2. (XLSX)

S7 File Raw data underlying Fig S3. (XLSX)

S8 File Raw data underlying Fig S4. (XLSX)

S9 File Raw data underlying Fig S5. (XLSX)

S10 File Raw data underlying Fig S6. (XLSX)
