# Supplementary figures and images for: Disruption of sulfur transferase complex increases bacterial intramacrophage persistence
Source: PLoS Pathog. 2025 May 14;21(5):e1013136. doi: 10.1371/journal.ppat.1013136 (PMC12077765; doi:10.1371/journal.ppat.1013136)

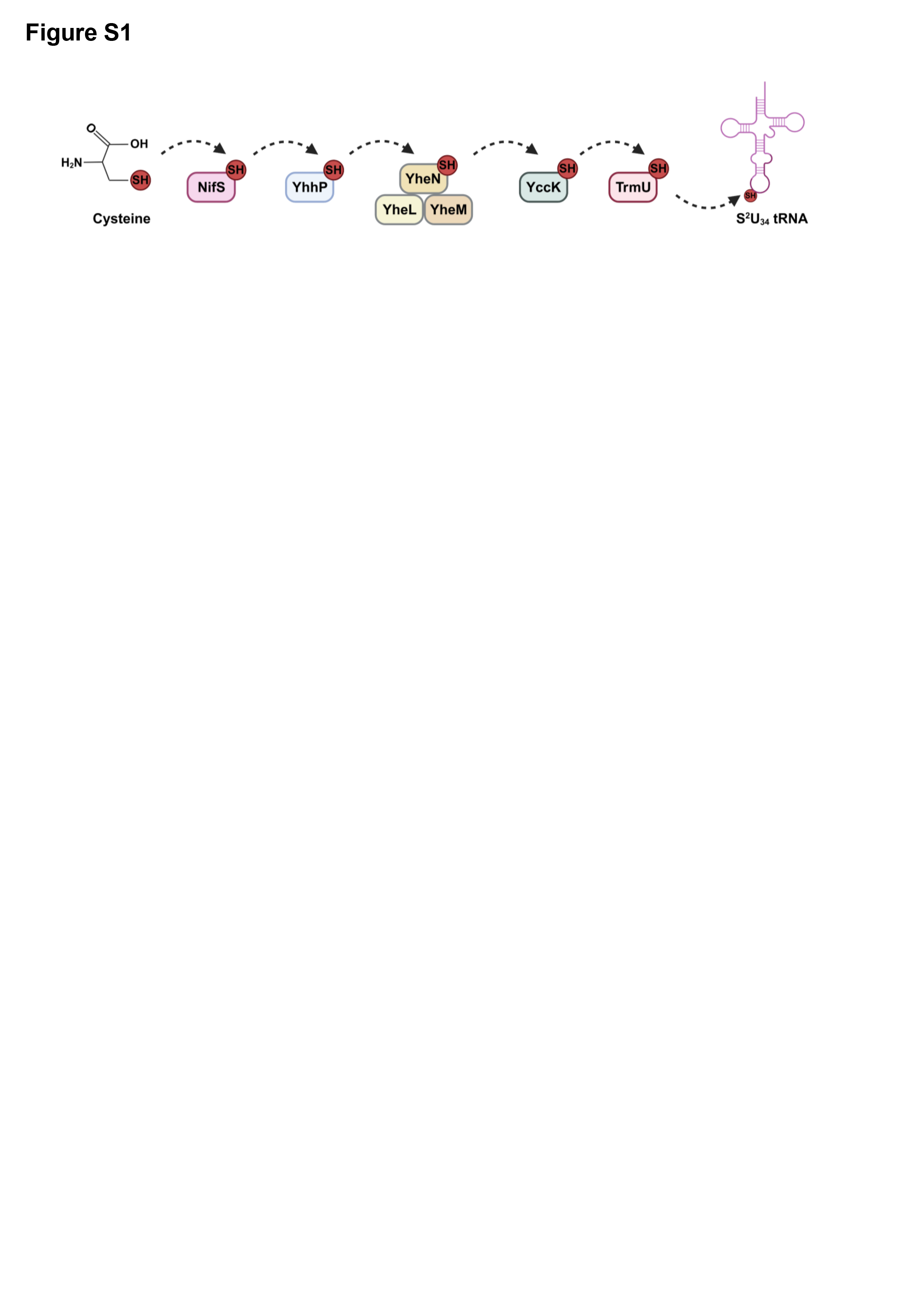

Supplement: S1 Fig — Created in BioRender. Tang, H. (2025) https://BioRender.com/u23k795. (TIF) [file ppat.1013136.s001.tif]

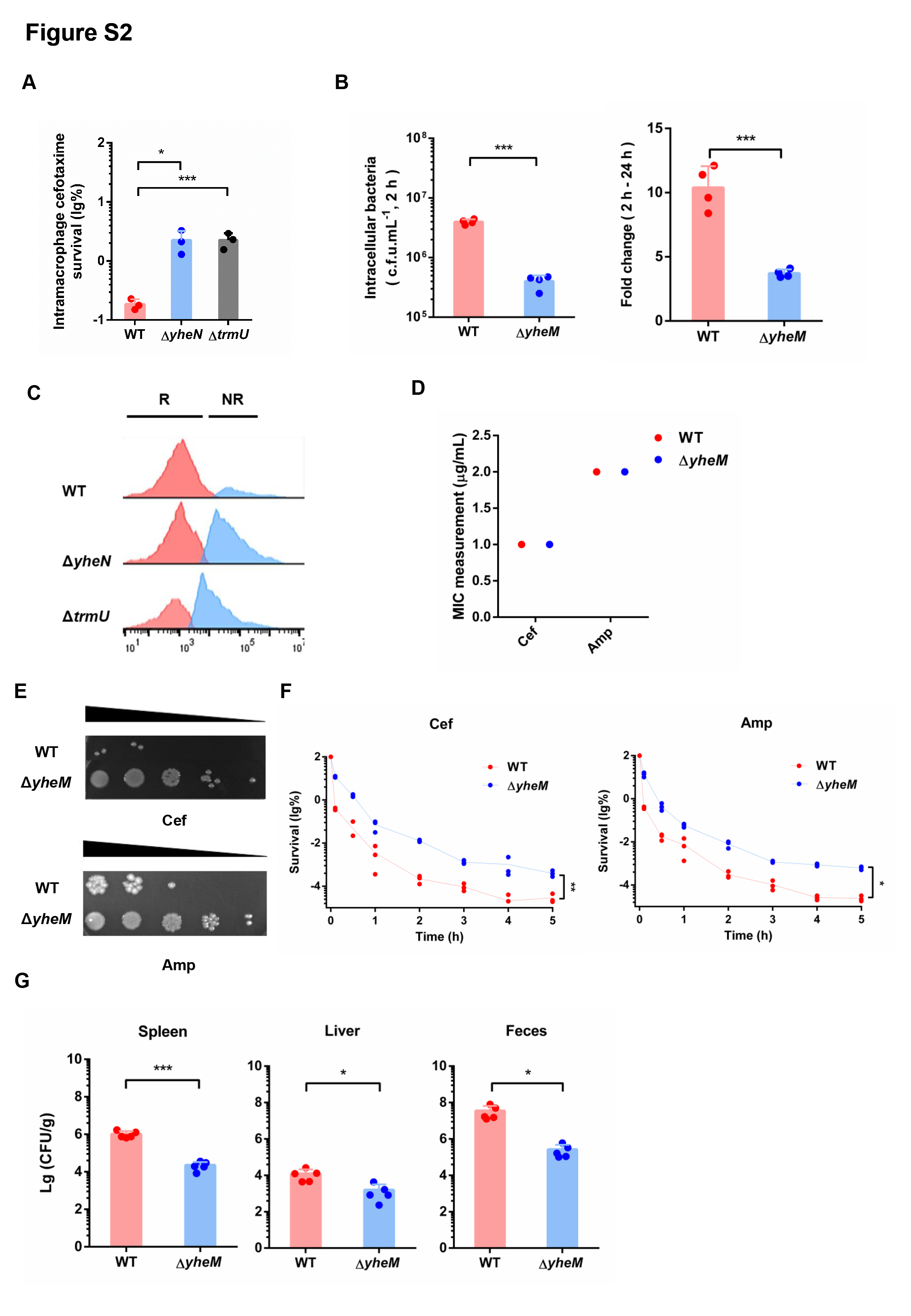

Supplement: S2 Fig — (A) Quantification of the percentage of surviving cells for wild-type strain and yheN deletion mutant and trmU deletion mutant in macrophages in the presence of cefotaxime for 24 hours. (B) Effects of yheM on Salmonella internalization (left) and replication (right) in macrophages. Macrophages were infected with wild-type strain or yheM deletion mutants. (C) Flow cytometry detection of green fluorescence in the wild-type strain and yheN deletion mutant and trmU deletion mutant labelled with CFSE. Representative fluorescence-activated cell sorting plots. (D) MIC tests of the wild-type strain and yheM deletion mutant with cefotaxime (Cef) and ampicillin (Amp). (E) Survival of the wild-type strain and yheM deletion mutant after exposure to 2 × MIC of cefotaxime (Cef) and ampicillin (Amp) for 2 hours. (F) Time-dependent killing curves of the wild-type strain and yheM deletion mutant in the exponential phase treated with ampicillin (left) or cefotaxime (right). (G) Bacterial load in tissues 24 hours after infection. Livers and spleens were harvested and the number of bacteria counted. Feces were collected and quantified at 24 hours post-infection. All experiments were performed in triplicate and one representative result is shown. Each point represents the result for one animal. Error bars represent the mean and standard deviation (SD) of four independent samples. *P < 0.05, **P < 0.01 and ***P < 0.001, Student’s t-test. (TIF) [file ppat.1013136.s002.tif]

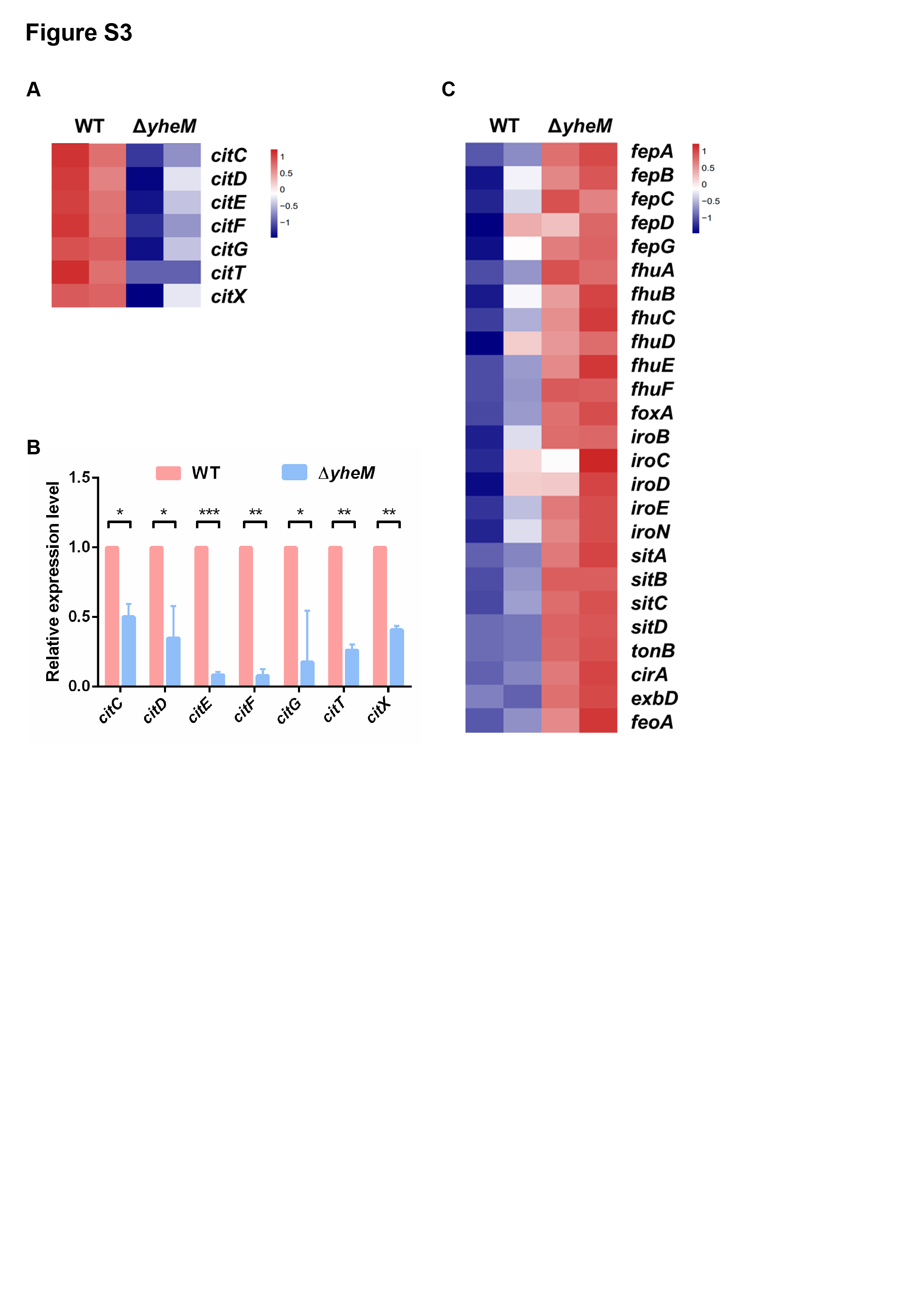

Supplement: S3 Fig — (A) Heat map based on RNA-seq results showing the relative transcription of genes involved in the citrate lyase and citrate transport regulon between the wild-type strain and the yheM deletion mutant. Cluster analysis was performed using the log10(FPKM+1) values and standardized by Z-score. (B) The relative expression level of the citrate regulon in the yheM deletion mutant (blue) compared to the expression in the wild-type strain (red) as determined by qRT-PCR. RNA was extracted from exponential phase Salmonella cultures. (C) Heat map based on RNA-seq results showing the relative transcription of genes involved in the iron transfer system between the wild-type strain and the yheM deletion mutant. Cluster analysis was performed using the log10(FPKM+1) values and standardized by Z-score. (TIF) [file ppat.1013136.s003.tif]

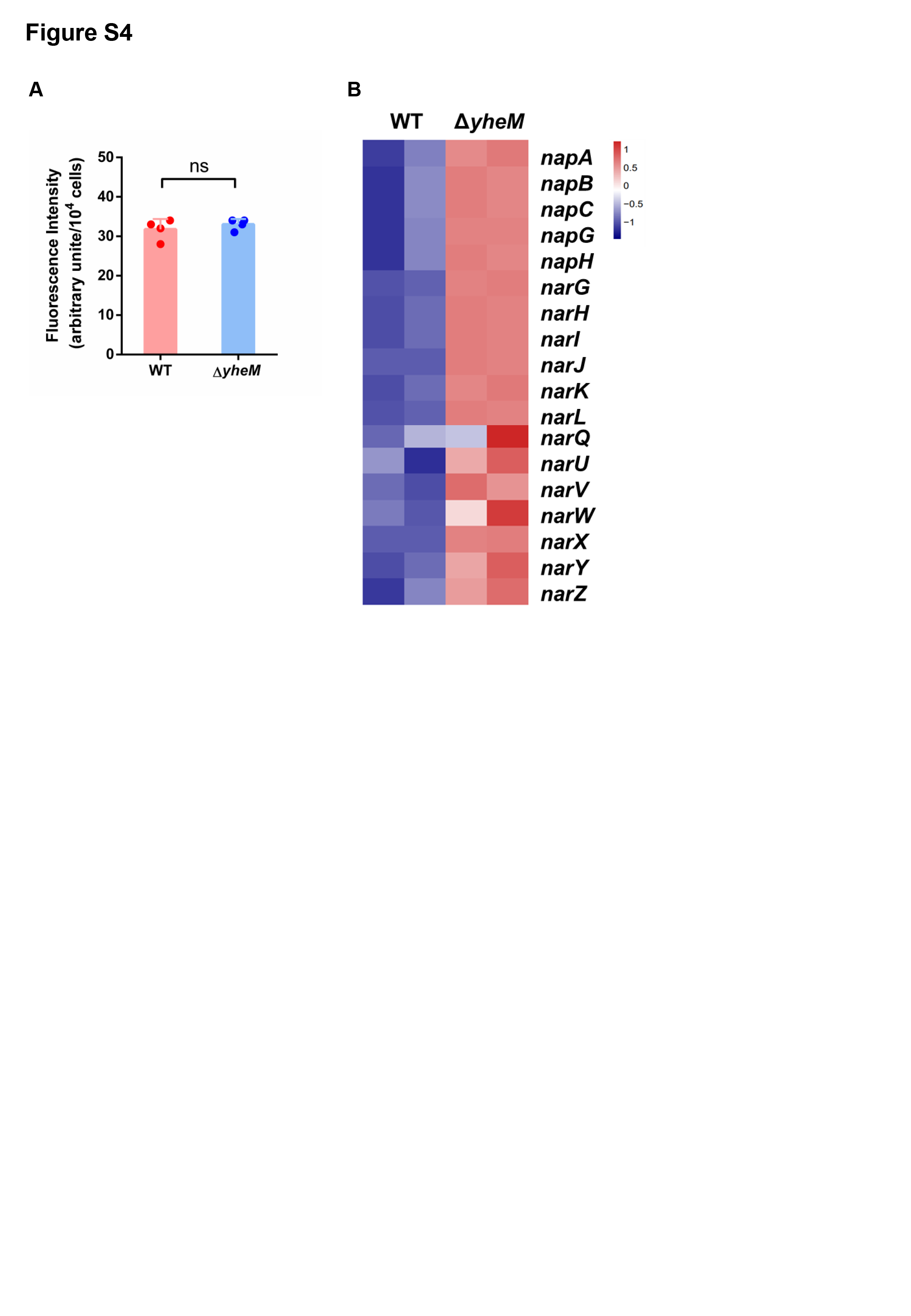

Supplement: S4 Fig — (A) ROS production in the wild-type strain and yheM deletion mutant in the exponential phase. The fluorescence signal of ROS was quantified at 488/525 nm (excitation/emission) (B) Heat map based on RNA-seq results showing the relative transcription of genes involved in the nitrogen metabolism regulon between the wild-type strain and the yheM deletion mutant. Cluster analysis was performed using the log10(FPKM+1) values and standardized by Z-score. (TIF) [file ppat.1013136.s004.tif]

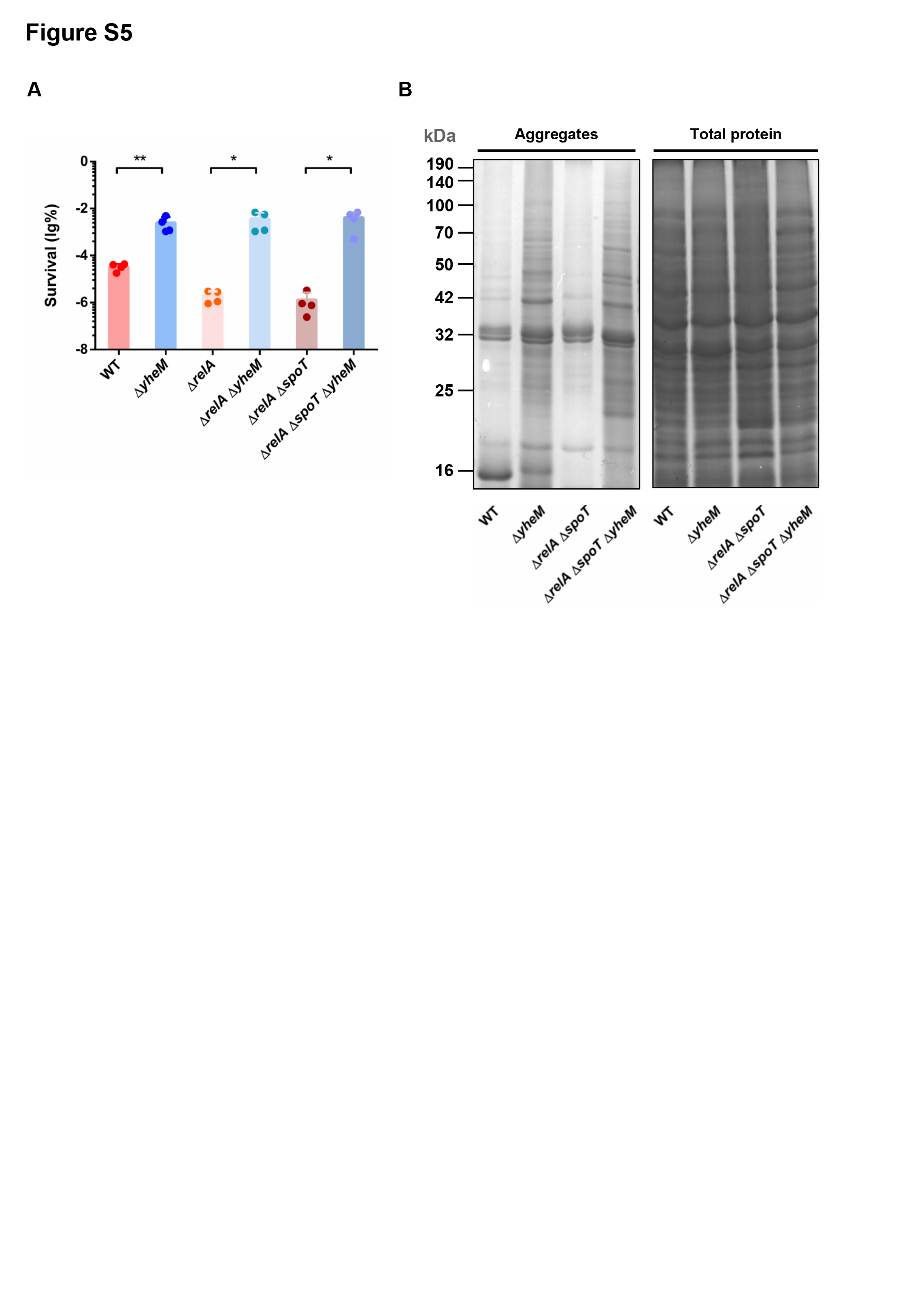

Supplement: S5 Fig — (A) Cefotaxime survival of the wild-type strain, yheM deletion mutant, relA deletion mutant, relA and yheM deletion mutant, relA and spoT deletion mutant and yheM, relA and spoT triple deletion mutant in the exponential phase. All experiments were performed in triplicate and one representative result is shown. Each point represents an independent sample. Error bars represent mean and standard deviation (SD). **P < 0.01; ***P < 0.001, Student’s t-test. (B) Protein aggregates isolated from the wild-type strain, yheM deletion mutant, relA and spoT double deletion mutant and yheM, relA and spoT triple deletion mutant in macrophages at 37°C. Total cell lysates were used to indicate identical protein concentrations between samples. (TIF) [file ppat.1013136.s005.tif]

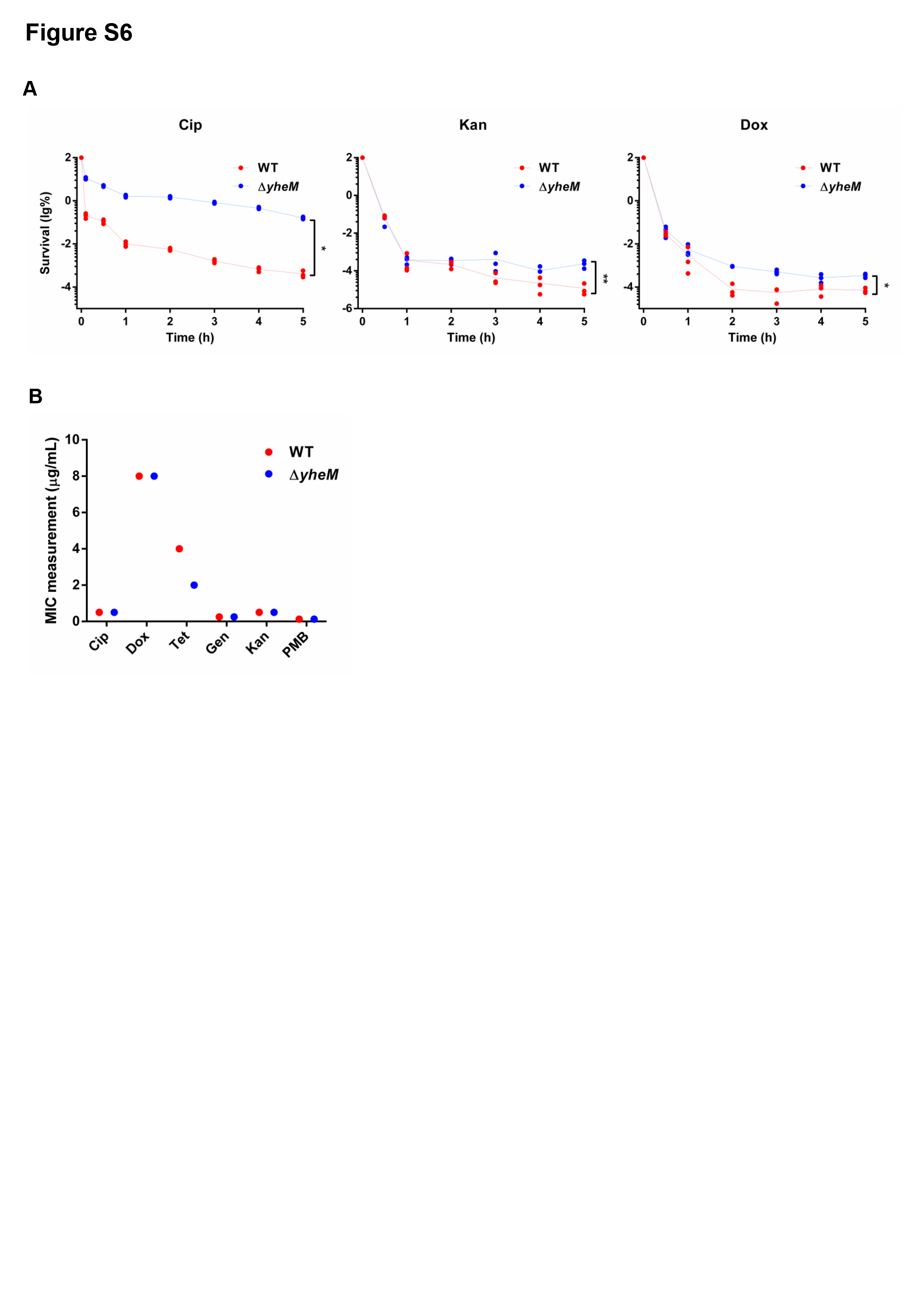

Supplement: S6 Fig — (A) Time-dependent killing curves of the wild-type strain and yheM deletion mutant treated with ciprofloxacin (200 μg/mL), doxycycline (200 μg/mL) and kanamycin (100 μg/mL). (B) MIC determination of the wild-type strain and yheM deletion mutant with ciprofloxacin (Cip), doxycycline (Dox), tetracycline (Tet), gentamicin (Gen), kanamycin (Kan) and polymyxin B (PMB). (TIF) [file ppat.1013136.s006.tif]
