# Supplementary material for: Disruption of sulfur transferase complex increases bacterial intramacrophage persistence
Source: PLoS Pathog. 2025 May 14;21(5):e1013136. doi: 10.1371/journal.ppat.1013136 (PMC12077765; doi:10.1371/journal.ppat.1013136)
Supplement: S1 Table — (DOCX) [file ppat.1013136.s007.docx]

**Supplementary materials**

**Supplementary tables**

**Table S1 Bacterial strains and plasmids used in this study.**

| **Strains and plasmids** | **Genotypes and characteristics** | **Sources or references** |
| --- | --- | --- |
| **Strains** |  |  |
| *E*. *coli* DH5α | F- 80lacZ M15 (lacZYA–argF) U169*eoR* *recA1* *endA1* *hsdR17* *phoA* *supE*44-thi-1 *gyrA96* *relA1* | Laboratory stock |
| *E*. *coli* BL21 | F- *ompT* *gal* *dcm* *lon* *hsdSB*(rB- mB-) λ(DE3 [*lacI* *lacUV*5-T7 gene 1 *ind1* *sam7* *nin5*]) | Laboratory stock |
| *S*. Typhimurium 14028S | Wild-type *S*. Typhimurium strain | Laboratory stock |
| Δ*yheM* | *S*. Typhimurium 14028S Δ*yheM* | Laboratory stock |
| Δ*yheN* | *S*. Typhimurium 14028S Δ*yheN* | This study |
| Δ*yheL* | *S*. Typhimurium 14028S Δ*yheL* | This study |
| Δ*yccK* | *S*. Typhimurium 14028S Δ*yccK* | This study |
| Δ*trmU* | *S*. Typhimurium 14028S Δ*trmU* | This study |
| Δ*yhhP* | *S*. Typhimurium 14028S Δ*yhhP* | This study |
| Δ*nifS* | *S*. Typhimurium 14028S Δ*nifS* | This study |
| Δ*relA* | *S*. Typhimurium 14028S Δ*relA* | This study |
| Δ*relA* Δ*spoT* | S. Typhimurium 14028S Δ*relA* Δ*spoT* | This study |
| Δ*yheM* Δ*relA* | S. Typhimurium 14028S Δ*yheM* Δ*relA* | This study |
| Δ*yheM* Δ*relA* Δ*spoT* | S. Typhimurium 14028S Δ*yheM* Δ*relA* Δ*spoT* | This study |
| **Plasmids** |  |  |
| pKD46 | Express λ red recombinase | Laboratory stock |
| pKD3 | Source for chloramphenicol resistance cassette | Laboratory stock |
| pBAD33 | Expression vector | Laboratory stock |
| p*yheM* | pBAD33 harboring *yheM* | This study |
