# Supplementary material for: Disruption of sulfur transferase complex increases bacterial intramacrophage persistence
Source: PLoS Pathog. 2025 May 14;21(5):e1013136. doi: 10.1371/journal.ppat.1013136 (PMC12077765; doi:10.1371/journal.ppat.1013136)
Supplement: S2 Table — (DOCX) [file ppat.1013136.s008.docx]

**Table S2** **Primers used in this study.**

| **Primer names** | **Sequences** |
| --- | --- |
| **Knock out** |  |
| *relA*-OUT-F | TTACCGGCTTACCGACTTCGGTAGGCCTGGTCCCTTAAGG  AGAGGACGGTGTAGGCTGGAGCTGCTTCG |
| *relA*-OUT-R | GCTTTGCTGAACGAGTAGCAAAGCCGCTACATGATTACTG  TCTGGGGTCATATGAATATCCTCCTTAGT |
| *spoT*-OUT-F | GCCGTTACCGCTATTGCTGAAGGTCGTCGTTAATCACAAA  GCGGGTCGCCCGTGTAGGCTGGAGCTGCTTCG |
| *spoT*-OUT-R | CTGGCGAGCATTTCGCATATACGCGCATAACGTTTTGGAT  TCATAGCGCATATGAATATCCTCCTTAGT |
| *trmU*-OUT-F | GGCTGCGTGATAGAATACGCCGCCTTGAAGTTCAATGTCG  CGAGTATTCCAGTGTAGGCTGGAGCTGCTTCG |
| *trmU*-OUT-R | CATCTTTTAAGCCGCTTTGAGGCGACTTGAATGGTGTTGTG  TATTGAATAACATATGAATATCCTCCTTAGT |
| *yheL*-OUT-F | TCCTGAGATTCTGAGGCGTTTGTGTAGGCTGGAGCTGCTTCG |
| *yheL*-OUT-R | CATATGAATATCCTCCTTAGTAAATATACAACGATCCCGCCA |
| *yheN*-OUT-F | CTTTCAGGGGCAGGATAAATAGTGTAGGCTGGAGCTGCTTCG |
| *yheN*-OUT-R | GACAAATGCAATACGTTTCATCATATGAATATCCTCCTTAGT |
| *yccK*-OUT-F | TTGATTACGCAGTGAAACGTTGTGTAGGCTGGAGCTGCTTCG |
| *yccK*-OUT-R | AACAGGCTTTAGTATTCGGTACATATGAATATCCTCCTTAGT |
| *yhhP*-OUT-F | TTTTCTACCATTGAGACGAAGGTGTAGGCTGGAGCTGCTTCG |
| *yhhP*-OUT-R | AGAATAGTATCTGCCGTGCAGCATATGAATATCCTCCTTAGT |
| *nifS*-OUT-F | AGCCGGTTGCCTGATTCCTTGCATTGAGTGATGTACGGAGTT  TAAGAGCAGTGTAGGCTGGAGCTGCTTCG |
| *nifS*-OUT-R | ATTACTTTTTCGCTGTAAGCCATTATAAATTCTCCTGATTCCG  ATACCGACATATGAATATCCTCCTTAGT |
| **qPCR** |  |
| qPCR-*citC*-F | GCGAAGGCTTAGCGTTAACG |
| qPCR-*citC*-R | CGACAAAGCGGTGTGTGATG |
| qPCR-*citD*-F | ATAAACCAGCTAGCCGTCGC |
| qPCR-*citD*-R | AGCGCCTTTATCATCGACGT |
| qPCR-*citE*-F | CCTGGAAGACTCCGTTGCAT |
| qPCR-*citE*-R | TCGTTGTTGGCGTCGGAATA |
| qPCR-*citF*-F | CGCAAACTGCTTGACGTTCA |
| qPCR-*citF*-R | TCGGTGAATTCGATAGGGCG |
| qPCR-*citX*-F | TTTCCTGAACTCGCCACCTG |
| qPCR-*citX*-R | AGCAGCGCCTCCATATGAAT |
| qPCR-*citG*-F | CACAAAGATATGGCGCTGGC |
| qPCR-*citG*-R | CGTTAAGCGACATCAGCAGC |
| qPCR-*yheL*-F | CCTTACCTCATTGCGCTTCG |
| qPCR-*yheL*-R | GTCAACCCTGACGACACTGT |
| qPCR-*yheN*-F | GCGCCTGGCAGAAATTGAAT |
| qPCR-*yheN*-R | TCTGCCGGCTTCAGTTTCAT |
| qPCR-*yheM*-F | ATTTGTCTTTTCCACCGCGC |
| qPCR-*yheM*-R | AAAGACGCCGTCGCTGATAA |
| qPCR-*yhhP*-F | CTCCCCTGACCATACGCTTG |
| qPCR-*yhhP*-R | TCGTCGGCGATAATCAGCAA |
| qPCR-*yccK*-F | CTCTGCGGAACACTGGGAAG |
| qPCR-*yccK*-R | CATTTTACCGGCTTGGGCAG |
| qPCR-*nifS*-F | GCTGATCATGGCGCTGAAAG |
| qPCR-*nifS*-R | TAGAGCTATGCGCCAGTTCG |
| qPCR-*trmU*-F | GGGCTCGACGGTAACAAAGA |
| qPCR-*trmU*-R | GATTCCCAGCCCTTTACGCT |
